# Pharmacological Effects and Development Prospects of the Main Active Compounds of *Paeonia* × *suffruticosa* Andrews in the Treatment of Panvascular Diseases

**DOI:** 10.3390/molecules31091514

**Published:** 2026-05-02

**Authors:** Xin-Wen Huang, Zhao-Yue Li, Fei-Yu Xie, Lin-Yu Chen, Xu Yang, Hui-Min Xiao, Si-Wang Wang

**Affiliations:** 1 Faculty of Life and Health Science, Northwest University, 229 Taibai Road, Xi’an 710069, China; 2 Shaanxi Key Laboratory of Natural Products & Chemical Biology, College of Chemistry & Pharmacy, Northwest A&F University, Yangling 712100, China

**Keywords:** *Paeonia* × *suffruticosa* Andrews, important active compounds, panvascular diseases, pharmacological effects, development application prospects

## Abstract

Panvascular diseases are complex, with systemic vascular system damage as the common pathological basis. The pathogenesis of panvascular diseases is closely related to vascular endothelial dysfunction, inflammatory responses, oxidative stress, abnormal lipid metabolism, platelet aggregation, and thrombosis, posing a serious threat to human health. The *Paeonia* × *suffruticosa* Andrews (*P. suffruticosa*), a type of medicinal peony, is one of the Standard Chinese medicinal herbs included in the Chinese Pharmacopoeia. The root bark, leaves, petals, pollen, seeds, and follicles of *P. suffruticosa* are rich in various active compounds, including paeonol, paeoniflorin, and α-linolenic acid. Modern studies have demonstrated that these compounds exhibit significant pharmacological activities, including vascular endothelial protection, lipid metabolism regulation, antiplatelet aggregation, and anti-inflammatory, antioxidant, and antithrombotic effects. Furthermore, their mechanisms of action are highly consistent with the key pathological processes of panvascular diseases, indicating that *P. suffruticosa* has important value in the prevention and treatment of such diseases. The information involved in the study was gathered from a variety of electronic resources, including PubMed, Web of Science, ScienceDirect, SciFinder, China National Knowledge Infrastructure (CNKI), and Google Scholar. The retrieval period was from 1999 to 2025. This review systematically summarizes the pharmacological effects of the main active compounds of *P. suffruticosa* on panvascular diseases, providing a theoretical reference for the in-depth development and utilization of *P. suffruticosa* resources and the development of innovative drugs for preventing and treating panvascular diseases.

## 1. Introduction

*Paeonia* × *suffruticosa* Andrews (*P. suffruticosa*), recorded as a traditional Chinese medicinal material in the Chinese Pharmacopoeia, has a long history of application for its root bark (Moutan Cortex, MC), which is known for its effects in clearing heat, cooling blood, promoting blood circulation, and resolving blood stasis [1]. However, modern research has revealed that the value of *P. suffruticosa* extends far beyond these traditional uses. The entire plant—including its leaves, petals, pollen, seeds, and follicles—is rich in a variety of unique bioactive compounds, demonstrating broad medicinal potential that surpasses conventional understanding [2,3].

**Figure 1:**
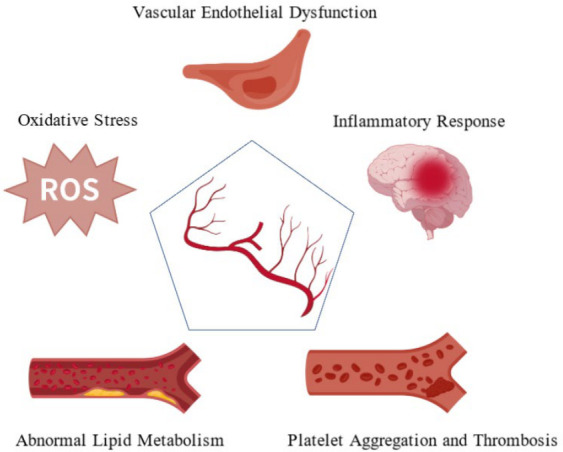
Five Key Factors in the Pathogenesis of Panvascular Diseases.

Panvascular diseases represent a complex group of disorders characterized by systemic vascular damage as a common pathological basis, encompassing conditions such as atherosclerosis, coronary heart disease, stroke, and peripheral vascular diseases. With persistently high morbidity and mortality rates, these diseases have become a major global public health challenge. The pathological mechanisms of panvascular diseases are intricate, with core processes including vascular endothelial dysfunction, chronic inflammation, oxidative stress, and lipid metabolism disorders, as well as platelet activation and thrombosis (Figure 1). These processes intertwine and reinforce one another, collectively driving the initiation and progression of the diseases [4,5].

Given the multi-target and multi-pathway nature of panvascular diseases, current chemical drugs targeting single pathways often exhibit limitations in prevention and treatment. Therefore, the search for lead compounds or active compounds from natural products with multi-target and synergistic effects has become an important direction in drug development and disease prevention strategies. As a “medicinal and edible” plant, *P. suffruticosa* contains various active compounds in its different parts, such as paeonol [6] and paeoniflorin in the root bark [1]; gallic acid, ellagic acid, and other phenols in the leaves [7,8]; anthocyanins, a subclass of flavonoids, in the petals [9,10]; and high levels of α-linolenic acid in the seed oil [11], as well as polysaccharides and cellulose in the follicles [12]. Numerous in vitro and in vivo studies have confirmed that these compounds precisely target the core pathological processes of panvascular diseases, demonstrating multiple pharmacological activities, including vascular endothelial protection, anti-inflammatory, antioxidant, lipid metabolism regulation, antiplatelet aggregation, and antithrombotic effects [11,13,14].

In recent years, studies on the active compounds and pharmacological effects of *P. suffruticosa* have increased; however, most have focused on single compounds (such as paeonol or paeoniflorin) [6,15] or one specific plant part for pharmaceutical use of *P. suffruticosa*, with MC as the dominant research object [1]. A comprehensive and systematic investigation focusing on whole-plant utilization and multi-compound synergy, as well as the correlation between *P. suffruticosa* active compound groups and panvascular disease pathogenesis, is still insufficient. This deficiency in systematic research greatly limits the intensive exploitation of *P. suffruticosa* resources and restricts the innovative drug research targeting the prevention and treatment of panvascular diseases.

Thus, this review was conducted to synthesize domestic and international research progress, systematically summarize the active compound characteristics of multiple plant parts serving as pharmaceutical raw materials of *P. suffruticosa*, including MC, leaves, petals, pollen, seeds, and follicles. Furthermore, it thoroughly analyzes the pharmacological mechanisms of key active compounds against panvascular diseases, focusing on vascular endothelial function, inflammation, redox balance, lipid metabolism and coagulation regulation. In addition, this review discusses the comprehensive utilization potential and innovative drug development prospects of *P. suffruticosa* resources, aiming to provide a theoretical basis and new perspectives for the deep exploitation of plant resources and the research of targeted therapeutic agents for panvascular diseases.

## 2. Research on the Major Plant Parts for Pharmaceutical Use of *P. suffruticosa*

### 2.1. Major Plant Parts for Pharmaceutical Use of P. suffruticosa

Moutan Cortex (MC) is one of the most commonly used decoction components in traditional Chinese medicine. MC is rich in active compounds, such as paeonol, paeoniflorin, and albiflorin, which yield substantial medicinal value [15,16]. Paeonol exhibits anti-inflammatory, antibacterial, antipyretic, and analgesic effects, demonstrating efficacy in pain relief and the treatment of inflammatory diseases [14,17,18]. Paeoniflorin exhibits multiple biological activities, including immune regulation, antioxidant activity, and antidepressant effects, thereby playing a positive role in maintaining human health [19,20].

*P. suffruticosa* leaves contain various active compounds and demonstrate unique medicinal potential worthy of in-depth research. Studies have shown that *P. suffruticosa* leaves are rich in compounds such as flavonoids, tannins, and amino acids. Among these compounds, flavonoids exhibit excellent antioxidant activity, effectively inhibiting lipid peroxidation and reducing free radical-induced cell damage. This property confers potential value in preventing vascular aging and protecting the cardiovascular system. Tannins have astringent and hemostatic effects, playing a role in the treatment of traumatic bleeding and other conditions. The various amino acids present provide nutrients and help maintain normal physiological metabolism [7,8].

*P. suffruticosa* petals are also noteworthy, as they are rich in flavonoids, polysaccharides, volatile oils, and other active compounds [10]. Flavonoids exhibit strong antioxidant capacity, scavenging free radicals and delaying cell senescence [21]; polysaccharide compounds play an important role in regulating immune function and enhancing the body’s resistance to pathogens [22]; and the volatile oils that give *P. suffruticosa* flowers a pleasant fragrance can also soothe the nerves and exert an aromatic resuscitating effect [23].

*P. suffruticosa* pollen, a natural medicinal resource, is also rich in active compounds and demonstrates medicinal value. This pollen contains abundant proteins, amino acids, vitamins, minerals, and bioactive compounds, such as flavonoids and sterols. Proteins and amino acids are essential bioactive compounds that provide nutritional support and promote tissue repair and growth. Vitamins and minerals play a crucial role in maintaining the body’s normal physiological functions [24,25].

*P. suffruticosa* seeds are also a highly valuable medicinal part. *P. suffruticosa* seed oil extracted through physical pressing, extraction, and other processes, is rich in unsaturated fatty acids, with an α-linolenic acid content as high as 45%. As an essential fatty acid for humans, α-linolenic acid is critical for blood lipid regulation, reducing blood viscosity, and preventing cardiovascular diseases; thus, it is of high nutritional and pharmacological value [11,26,27].

*P. suffruticosa* follicles, an integral part of the plant’s reproductive organs, also possess potential medicinal value. Modern studies have shown that *P. suffruticosa* follicles are rich in polysaccharides, cellulose, flavonoids, and other active compounds [12,28,29]. Among these active compounds, follicle polysaccharides, as natural macromolecular compounds, exhibit significant immune-enhancing activity and can strengthen the body’s immune response by promoting the macrophage phagocytic functions and regulating cytokine secretion [30,31]. Flavonoids, another key component in follicles, exhibit both antioxidant and anti-inflammatory effects due to their structural diversity, in that they (1) protect vascular endothelial cells from oxidative damage by scavenging oxygen free radicals and (2) can inhibit the overexpression of inflammatory factors [32,33,34], thereby improving the inflammatory state of blood vessel walls. This mechanism is closely related to the core vascular wall lesion pathology observed in panvascular diseases.

*P. suffruticosa* MC, leaves, petals, pollen, seeds, and follicles provide numerous benefits to human health and demonstrate the importance of *P. suffruticosa* in the medical field. Recent studies have indicated that the active compounds in the pharmaceutically relevant plant parts of *P. suffruticosa* play a positive role in regulating vascular function and improving cardiovascular metabolism, which is closely related to the prevention and treatment of panvascular diseases [35,36,37,38,39,40].

### 2.2. Studies on the Specifications of the Major Plant Parts for Pharmaceutical Use of P. suffruticosa

Table 1 systematically presents the medicinal specifications, identification and preparation processes, and corresponding provincial, Pharmacopoeia, or journal standards of the major plant parts for pharmaceutical use of *P. suffruticosa* (including MC and its different processing specifications, as well as *P. suffruticosa* leaves, petals, pollen, seeds, and follicles). This provides an intuitive reference for research on the standard system of the major plant parts for pharmaceutical use of *P. suffruticosa*.

## 3. Main *P. suffruticosa* Active Compounds and Their Contents

### 3.1. Moutan Cortex

Among the plant parts for pharmaceutical use of *P. suffruticosa*, the active compounds of MC, the core and most commonly used part, have been studied systematically to the greatest depth, confirming the presence of various active compounds.

#### 3.1.1. Monoterpene Glycosides

Monoterpene glycosides are a crucial class of active compounds in MC, with paeoniflorin serving as a representative. The Chinese Pharmacopoeia (2025 Edition) clearly stipulates that the content range of paeoniflorin in MC is 9.0–17.0 mg/g [1]. Specific research data collected from the same batch of medicinal materials demonstrated a paeoniflorin content of 9.01 mg/g. MC also contains other monoterpene glycoside compounds, such as oxypaeoniflorin (with a measured content of 0.65 mg/g) [65]. Together, these compounds constitute one of the material bases for the pharmacological effects of MC.

#### 3.1.2. Phenols

Phenols are the characteristic compounds of MC, among which paeonol is the most well-studied. The Chinese Pharmacopoeia stipulates that the content of paeonol in MC shall not be less than 12 mg/g [1]. One study demonstrated a paeonol content of 25.23 mg/g in one batch of medicinal materials; another revealed an average content of 14.901 mg/g. MC also contains phenols, such as gallic acid (1.38 mg/g) and ellagic acid. The above compounds synergistically participate in the pharmacological effects of MC [65,66].

#### 3.1.3. Other Compounds

In addition to the main categories of compounds mentioned above, MC also contains various other compounds, including triterpenoids (e.g., oleanolic acid), flavonoids (e.g., quercetin), and volatile oils. Across relevant research reports, over 163 compounds have been isolated and identified from MC. The average content of quercetin in MC is 0.06 mg/g, further reflecting the MC’s diverse and complex medicinal value [66,67].

### 3.2. P. suffruticosa Leaves

The medicinal value of aboveground *P. suffruticosa* leaves has long been overlooked; however, recent studies have demonstrated that these leaves contain numerous active compounds, including phenols, glycosides, flavonoids, polysaccharides, amino acids, vitamins, and fatty acids, indicating the potential for development and utilization.

#### 3.2.1. Phenols

Phenols, primarily gallic acid, ellagic acid, and 1,2,3,4,6-O-pentagalloylglucose, are a well-researched class of active compounds present at high content levels in *P. suffruticosa* leaves.

Across 10 *P. suffruticosa* leaf sample batches from different sources, the average gallic acid content was found to be 1.65 mg/g; furthermore, its content increases significantly after enrichment via extraction processes, with an average reaching 61.14 mg/g in one multiple-batch assessment. Ellagic acid is present at the highest content level in *P. suffruticosa* leaves, with an average content across 10 sample batches reaching 50.58 mg/g. The average content of 1,2,3,4,6-O-pentagalloylglucose in *P. suffruticosa* leaves is 6.06 mg/g [59,68].

Significant varietal differences exist in the total phenol content of *P. suffruticosa* leaves. Among 20 *P. suffruticosa* leaf varieties from Luoyang, the total phenol content was found to range from 68.31 to 188.19 mg/g, whereas the Haihuang variety demonstrated the highest content at 188.19 mg/g. Moreover, the total phenol content exhibits a significant positive correlation with antioxidant activity, serving as an important indicator for *P. suffruticosa* leaf antioxidant capacity assessments [69].

#### 3.2.2. Monoterpene Glycosides

The primary monoterpene glycoside in *P. suffruticosa* leaves is paeoniflorin, with an average content of 7.80 mg/g observed across 10 sample batches [8,59]. Further studies have revealed significant differences in paeoniflorin content among different *P. suffruticosa* leaf varieties. Among 20 *P. suffruticosa* leaf varieties from Luoyang, the content of paeoniflorin was found to range from 20.50 to 45.10 mg/g, with the Haihuang variety demonstrating the highest content (45.10 mg/g) [69]. The paeoniflorin content in *P. suffruticosa* leaf extract is 39.83 mg/g; the difference between this value and that in the raw material may be attributable to extraction processes or varietal characteristics [68].

#### 3.2.3. Flavonoids

*P. suffruticosa* leaves are rich in diverse flavonoids, mainly rhoifolin, apigenin-7-O-β-D-glucoside, luteolin, kaempferol, and apigenin. It has been demonstrated that the average rhoifolin and apigenin-7-O-β-D-glucoside contents in *P. suffruticosa* leaves are 1.62 mg/g and 1.42 mg/g, respectively [59].

Regarding other flavonoid compounds, one study revealed that the total flavonoid content in 20 *P. suffruticosa* leaf varieties from Luoyang ranged from 20.71 to 54.03 mg/g, with the Yaohuang variety demonstrating the highest content (54.03 mg/g) [69]. The average kaempferide, quercetin, and isorhamnetin contents in *P. suffruticosa* leaf extract are 21.73, 31.92, and 20.18 mg/g; thus, extraction further enriches the flavonoid compounds [68].

#### 3.2.4. Other Compounds

In addition to the above main active compounds, *P. suffruticosa* leaves contain various nutritional and functional components, further reflecting their diverse development value.

For polysaccharides, one study demonstrated a mass fraction of total polysaccharides ranging from 1.40 to 1.95 mg/g in dried *P. suffruticosa* leaves from 6 different producing areas [70].

Regarding basic nutritional components, *P. suffruticosa* leaves generally contain protein (approximately 150 mg/g), soluble sugar (approximately 120 mg/g), and fat (approximately 40 mg/g) [8]. The protein content of *P. suffruticosa* leaves from Yuncheng reaches as high as 219 mg/g [71]. These leaves are rich in amino acids, including multiple essential amino acids such as isoleucine, leucine, and lysine. The essential and medicinal amino acid contents in *P. suffruticosa* leaves from Yuncheng County have been found to be 80.1 mg/g and 126.1 mg/g, respectively, with an essential amino acid index (EAAI) of 0.79, indicating the potential of *P. suffruticosa* leaves as a high-quality protein source [71].

In terms of vitamins, *P. suffruticosa* leaves contain vitamin A and vitamin C; the vitamin C content in *P. suffruticosa* leaves from Caoxian County has been found to reach 0.548 mg/g [71]. Furthermore, *P. suffruticosa* leaves mainly contain myristic (C14:0), palmitic (C16:0), oleic (C18:1), stearic (C18:0), linoleic (C18:2), and α-linolenic acids (C18:3) as fatty acids. Among these, α-linolenic acid, an essential unsaturated fatty acid in humans, possesses important nutritional and health-preserving value [72]. Additionally, there are differences in the total fatty acid content of *P. suffruticosa* leaves from different producing areas, with the total fatty acid content in those from Chengwu County reaching 4.4 mg/g [71].

### 3.3. P. suffruticosa Petals

*P. suffruticosa* petals contain various active compounds, such as phenols, flavonoids, monoterpene glycosides, anthocyanins, and essential oil, and are rich in proteins, vitamins, and amino acids; thus, they contain abundant material and are of high nutritional value.

#### 3.3.1. Phenols

Phenols, primarily gallic acid and its derivatives and tannins, demonstrate high content levels in *P. suffruticosa* petals. Studies have shown that the content of gallic acid in freeze-dried ultrafine powder of *P. suffruticosa* petals ranges from 4.29 to 5.00 mg/g [60]. In one study, two gallic acid tannin compounds, namely 1-O-galloyl-β-D-glucose and 1,2,3,4,6-pentagalloyl-β-D-glucose, were isolated from the petals of *Paeonia ostii* (Fengdan *P. suffruticosa*) for the first time [73].

#### 3.3.2. Flavonoids

*P. suffruticosa* petals are rich in diverse flavonoids, mainly rutin, quercetin, kaempferol, luteolin, and apigenin. The content levels of these flavonoids are closely related to petal color and developmental stage.

Rutin and apigenin account for a relatively high proportion of total polyphenols in 11 *P. suffruticosa* varieties. Among them, the apigenin content in the Shouanhong variety reaches 17.01 mg/g, and the rutin content in Yinhong Qiaodui reaches 16.51 mg/g [74].

The total flavonoid content varies significantly across petals of different colors and developmental stages, with the highest content observed at the round bud stage (S1). A gradual decrease is observed with flower development. The total flavonoid content of dark-colored varieties (e.g., the purple Gejinzi variety) is significantly higher than that of light-colored varieties. The total flavonoid content of Gejinzi reaches 52.81 mg/g at the full bloom stage (S4) [9]. In addition, flavonoid glycosides, such as kaempferol-3,7-di-O-β-D-glucoside, have been isolated from the petals of *Paeonia ostii*, further demonstrating the diversity of flavonoid compounds [73].

#### 3.3.3. Monoterpene Glycosides

Monoterpene glycosides are the characteristic compounds of *P. suffruticosa* petals. Paeoniflorin and its derivatives have been detected in freeze-dried ultrafine *P. suffruticosa* petal powder, with a paeoniflorin content ranging from 1.27 to 1.81 mg/g and a hydroxypaeoniflorin content ranging from 2.13 to 3.32 mg/g.

*P. suffruticosa* petals also contain other monoterpene glycoside compounds, such as rhoifolin (2.92–3.75 mg/g), further demonstrating enrichment with this class of compounds [60].

#### 3.3.4. Anthocyanins

Anthocyanins are the key pigment compounds regulating *P. suffruticosa* petal color, primarily cyanidin, pelargonidin, peonidin, and their derivatives. Anthocyanin content is significantly positively correlated with petal color depth.

Regarding content and distribution characteristics, the anthocyanin content of pink, red, and purple petals is significantly higher than that of white and yellow varieties. In the purple Shouanhong variety, the cyanidin content reaches 6.03 mg/g, and that of pelargonidin reaches 3.96 mg/g, which is 80 and 12 times that observed in pink and red varieties, respectively [74]. Metabolomic analyses have demonstrated that in dark-colored varieties such as Gejinzi and Luoyanghong, the contents of anthocyanin compounds such as peonidin-3,5-O-di-β-glucoside and petunidin-3-glucoside are significantly increased; the content of peonidin-3,5-O-di-β-glucoside in Gejinzi is 218 times that of Fengdanbai (white *Paeonia ostii*) at the full bloom stage (S4) [9].

#### 3.3.5. Other Compounds

Essential oils (EOs) are characteristic constituents of *P. suffruticosa* petals. GC-MS analysis has identified abundant terpenes, alcohols, esters, and aromatic compounds in *P. suffruticosa* petal EOs, which collectively contribute to their unique floral aroma. Different varieties exhibit distinct essential oil compositions: for example, *Paeonia delavayi* EOs contain 194 compounds, dominated by alcohols, aldehydes, ketones, and esters. *P. suffruticosa* petal EOs also exhibit significant antioxidant activity, with great potential for applications in aromatherapy, functional cosmetics, and natural food flavoring [75,76].

*P. suffruticosa* petals are rich in various nutrients, including basic nutrients, vitamins, and amino acids.

Regarding basic nutrients, the freeze-dried ultrafine powder of *P. suffruticosa* petals contains 150 mg/g of protein and 19 mg/g of fat; the contents of soluble sugar and titratable acid increase gradually with flower development; the soluble sugar content of Fengdanbai reaches 45.32 mg/g at the full bloom stage (S4) [9,60].

Regarding vitamins and amino acids, the vitamin C content in *P. suffruticosa* petals reaches as high as 70.45 mg/g, much higher than that of common fruits and vegetables; among the 18 amino acids in *P. suffruticosa* petals, 8 are essential amino acids in humans (including threonine and valine), and glutamic acid content reaches 14.2 mg/g [60].

### 3.4. P. suffruticosa Pollen

The male reproductive cell, *P. suffruticosa* pollen, contains active compounds such as polyphenolic substances (predominantly flavonoids), polysaccharides, and saponins. Furthermore, the pollen contains abundant proteins, amino acids, vitamins, and mineral elements.

#### 3.4.1. Flavonoids

Regarding total flavonoids, assessments have shown that the total flavonoid content of *P. suffruticosa* pollen is 14.94 mg/g; this value is significantly higher than those of common plant pollens, such as rapeseed and camellia [24,77]. The specific compounds of *P. suffruticosa* pollen include rutin, luteolin glycoside, and quercetin, among which rutin accounts for a significant proportion of the flavonoid content. In addition, flavonoid glycosides such as limocitrin-3-O-β-D-sophoroside have been isolated from the pollen of *Paeonia rockii* (Ziban *P. suffruticosa*), further demonstrating the diversity of flavonoid compounds in *P. suffruticosa* pollen [78].

#### 3.4.2. Other Compounds

In addition to the main active compounds, *P. suffruticosa* pollen contains various nutritional and functional components, further reflecting its diverse development value.

Studies have shown that the total polysaccharide content in *P. suffruticosa* pollen reaches 171.08 mg/g, and the total saponin content is 71.66 mg/g [24]. Moreover, *P. suffruticosa* pollen is rich in various nutrients, including basic nutrients, amino acids, vitamins, and mineral elements, and has high edible and health care value.

Regarding basic nutrients and amino acids, an analysis of *Paeonia ostii* pollen demonstrated a protein content of approximately 15; furthermore, 18 amino acids were observed, including 8 essential amino acids in humans, such as threonine and valine. Among these amino acids, glutamic acid exhibits the highest content level and can participate in metabolic regulation [77]. Regarding vitamins and mineral elements, *P. suffruticosa* pollen is rich in vitamin E and B vitamins, among others; the content of vitamin E reaches 32 mg/g [77]. The mineral elements in this pollen include potassium (1.20 mg/g), calcium (0.85 mg/g), zinc (0.025 mg/g), and selenium (0.00015 mg/g) [24].

### 3.5. P. suffruticosa Seeds

*P. suffruticosa* seeds are critical for reproduction; thus, traditional studies primarily focused on agronomic traits. In contrast, modern studies have revealed that the oil constituents of these seeds possess outstanding nutritional and health care value. *P. suffruticosa* seeds exhibit a high oil content (300–400 mg/g), and the seed oil extracted from them is rich in active compounds, such as unsaturated fatty acids, sterols, and vitamins, showing significant development potential as functional foods, nutritional supplements, and medicine.

#### 3.5.1. Oil Constituents

The core active constituents of *P. suffruticosa* seeds is oil, characterized by its rich and balanced composition of unsaturated fatty acids.

The essential ω-3 fatty acid α-linolenic acid is the primary compound of *P. suffruticosa* seed oil. Studies have shown that the α-linolenic acid content in grade I *P. suffruticosa* seed oil is ≥420 mg/g, and that in grade II is ≥ 380 mg/g; among different varieties, the α-linolenic acid content of *Paeonia ostii* seed oil is the highest (452–526 mg/g), significantly higher than that of walnut (100–150 mg/g) and olive (<10 mg/g) oil [64].

The content of linoleic acid (ω-6) is ≥250 mg/g, and that of oleic acid (ω-9) is ≥210 mg/g; the above three unsaturated fatty acids form a reasonable ratio (approximately 1:1.5:0.5) and can synergistically regulate lipid balance [64]. In addition, *P. suffruticosa* seed oil contains a small concentration of saturated fatty acids, such as palmitic acid (<100 mg/g) and stearic acid (<30 mg/g), further improving its oxidative stability [63].

#### 3.5.2. Unsaponifiable Compounds

The unsaponifiable fraction of *P. suffruticosa* seed oil is rich in bioactive compounds, providing a significant material basis for its potential healthcare functions. The total sterol content is ≥1.8 mg/g, mainly including β-sitosterol (accounting for 70–80%), stigmasterol (10–15%), and campesterol (5–8%) [64]. The total vitamin E content is ≥0.3 mg/g, mainly comprising α-tocopherol (~60%) and γ-tocopherol (~30%) [63].

#### 3.5.3. Other Compounds

In addition to oil constituents, *P. suffruticosa* seeds contain various non-lipophilic compounds with potential development and utilization value. The protein content in defatted meal is 250–300 mg/g, which contains 17 amino acids, 35% of which are essential amino acids, such as lysine and threonine; thus, this compound can be developed and utilized as a plant protein resource [77]. The seed embryo contains flavonoid compounds, including rutin and quercetin, with a total content of approximately 0.8–1.2 mg/g [63].

### 3.6. P. suffruticosa Follicles

*P. suffruticosa* follicles, the fruit’s outer shell, were previously regarded as a by-product of cultivation; however, systematic studies investigating their active compounds have demonstrated richness in various functional compounds, such as phenols, flavonoids, and polysaccharides; thus, *P. suffruticosa* follicles demonstrate potential value in natural product development and resource recycling.

#### 3.6.1. Phenols

Phenols are the active compounds with the highest content in *P. suffruticosa* follicles, represented by gallic acid and ellagic acid. The content levels of these phenols are stable, and the detection methods available are mature and reliable; thus, these compounds form the core material basis for the physiological functions of *P. suffruticosa* follicles. An analysis of 12 batches of *P. suffruticosa* follicle samples revealed a gallic acid content range of 7.73–10.00 mg/g and an ellagic acid content range of 11.31–12.50 mg/g [28].

#### 3.6.2. Flavonoids

Flavonoids in *P. suffruticosa* follicles, primarily luteolin and apigenin, further enrich the composition of their active compounds. Detection via an optimized extraction process revealed luteolin and apigenin contents of 0.151 mg/g and 0.104 mg/g, respectively [79].

#### 3.6.3. Other Compounds

In addition to phenols and flavonoids, *P. suffruticosa* follicles contain active compounds such as polysaccharides and cellulose, which have unique functional value. Researchers have systematically analyzed the chemical composition and biological activity of *P. suffruticosa* pod polysaccharides. A monosaccharide composition analysis revealed the presence of mannose, rhamnose, glucuronic acid, galacturonic acid, glucose, galactose, and fucose, with a molar ratio of 1.44:2.87:0.32:18.99:3.99:10.96:1.85. Regarding composition characteristics, the polysaccharide content is acidic, with galacturonic acid and galactose as the main compounds [80].

*P. suffruticosa* follicles exhibit a high cellulose content (approximately 300–400 mg/g) and stable structure; after pretreatment, they can be used as dietary fiber raw materials or precursors for biodegradable materials, demonstrating potential application value as functional food additives and environmentally friendly packaging materials [81].

## 4. TCM, Modern Medicine Concepts, and Panvascular Disease Mechanistic Research

### 4.1. TCM and Modern Medicine Concepts of Panvascular Diseases

The term “panvascular diseases” was first proposed by Peter Lanzer and Eric J. Topol in 2002, defined as a group of systemic diseases with damage to the systemic vascular system as the common pathological basis. The term covers single vascular bed lesions (such as atherosclerosis, coronary heart disease, and cerebrovascular diseases) and diseases involving multiple vascular beds [82,83,84]. The core mechanisms of panvascular diseases are vascular endothelial dysfunction, inflammatory responses, oxidative stress, abnormal lipid metabolism, platelet aggregation, and thrombosis. With population aging and lifestyle changes, panvascular diseases have become a significant contributor to persistently high global mortality rates [85].

From the perspective of traditional Chinese medicine (TCM) theory, the pathogenesis of panvascular diseases is primarily characterized by “deficiency in root and excess in branch, qi deficiency and blood stasis”. The theories of “stasis-toxin” [86] and “turbid stasis damaging vessels” [87] hold that turbid stasis is both a cause and a pathogenesis of the disease. Clinically, the core treatment principle is often “promoting blood circulation and detoxification,” which stabilizes the vascular internal environment through anti-inflammation and anti-oxidative stress, embodying the TCM concept of “preventive treatment of disease” (zhi wei bing) [88,89,90].

### 4.2. Panvascular Disease Mechanistic Research

Panvascular diseases are a group of diseases that involve the systemic vascular system, including atherosclerosis, coronary heart disease, stroke, and peripheral vascular disease. Their occurrence and development involve complex processes, with interactions between multiple factors and mechanisms [4,5]. Accordingly, an in-depth understanding of the pathogenesis of panvascular diseases is crucial for their prevention, diagnosis, and treatment. The following sections provide an in-depth examination of the pathogenesis of panvascular diseases, focusing on vascular endothelial dysfunction, inflammatory response, oxidative stress, abnormal lipid metabolism, and platelet aggregation and thrombosis (Figure 1).

#### 4.2.1. Vascular Endothelial Dysfunction Is the Initiating Factor in Disease Onset

Vascular endothelial cells form a single layer of cells that line the inner walls of blood vessels. These cells act as a crucial barrier for maintaining vascular homeostasis and perform several important functions, including regulating vascular relaxation and contraction, inhibiting platelet aggregation, and exerting anti-inflammatory and antithrombotic effects [91]. Vascular endothelial dysfunction, manifesting as impaired vascular relaxation function and increased permeability, among other effects, is the initiating factor in the development of panvascular disease [92].

Vascular endothelial dysfunction first leads to abnormal vascular relaxation, characterized by reduced nitric oxide (NO) production and enhanced vascular responsiveness to vasoconstrictors. This effect increases the likelihood of vascular spasm, thereby affecting the blood supply to tissues and organs. Meanwhile, increased endothelial cell permeability allows lipids, inflammatory cells, and other blood-borne bioactive mediators to more easily enter the vascular wall, triggering a cascade of reactions, including platelet adhesion and inflammatory factor infiltration. Adhered platelets and infiltrated inflammatory cells release various bioactive mediators (e.g., growth factors and cytokines) that further stimulate vascular smooth muscle cell proliferation and migration. This effect results in vascular wall thickening and remodeling, ultimately promoting the onset of atherosclerosis [93,94,95].

#### 4.2.2. Inflammatory Response Is the Core Driver Throughout the Disease Course

The inflammatory response plays a pivotal role throughout the entire process of panvascular diseases, including occurrence, development, and associated complications. Chronic inflammation of the vascular wall serves as a critical basis for the initiation and progression of atherosclerosis, as it can induce endothelial cell damage, rendering the originally smooth vascular endothelium rough and further attracting the infiltration of monocytes and lymphocytes. These infiltrated immune cells transform into foam cells within the vascular wall, and the massive accumulation of foam cells forms a lipid core, accelerating the progression of atherosclerotic plaques [96,97].

Specifically, when the body is stimulated by pathogen-associated molecular patterns (PAMPs) such as lipopolysaccharide (LPS), the nuclear factor-κB (NF-κB) pathway is activated. NF-κB is a crucial transcription factor; once activated, it translocates into the cell nucleus and promotes the expression of genes encoding inflammatory factors, such as interleukin-6 (IL-6) and tumor necrosis factor-α (TNF-α), resulting in the massive release of these inflammatory factors. These inflammatory factors further damage vascular endothelial cells, increasing their permeability and facilitating the entry of more inflammatory cells and lipids into the vascular wall; furthermore, they exacerbate vascular smooth muscle cell proliferation and migration, resulting in vascular wall thickening and remodeling, which further aggravates vascular damage [98,99,100].

#### 4.2.3. Oxidative Stress Is a Key Inducer of Vascular Damage

Reactive oxygen species (ROS) and reactive nitrogen species (RNS) are highly reactive molecules produced during cellular metabolism. Under normal physiological conditions, the body’s antioxidant system can maintain the balance between the production and scavenging of ROS and RNS. However, when the body is in a state of oxidative stress, this balance is disrupted, leading to their excessive accumulation [101,102,103].

Excessive ROS and RNS can directly induce damage to vascular endothelial cells, disrupting their structure and function, and causing an imbalance in the secretion of vasoactive mediators. For instance, the production of NO decreases, while the production of vasoconstrictive mediators such as Endothelin-1 (ET-1) increases, resulting in impaired vascular relaxation function. Meanwhile, oxidative stress promotes the oxidative modification of low-density lipoprotein (LDL) to form oxidized low-density lipoprotein (ox-LDL). Ox-LDL exhibits a stronger atherogenic effect: it can be taken up by monocytes to form foam cells, and simultaneously triggers inflammatory responses that attract more immune cell infiltration. In addition, oxidative stress induces lipid peroxidation, which damages the lipid and protein structures of the vascular wall, further impairs the integrity of the vascular wall, and promotes the progression of atherosclerosis [104,105].

#### 4.2.4. Abnormal Lipid Metabolism Serves as the Material Basis for Atherosclerotic Plaque Formation

Elevated serum total cholesterol (TC), triglycerides (TG), low-density lipoprotein cholesterol (LDL-C), and decreased high-density lipoprotein cholesterol (HDL-C) are key risk factors for panvascular diseases. LDL-C is the main carrier that transports cholesterol from the liver to peripheral tissues. When the level of LDL-C in the blood increases, excess LDL-C enters the vascular wall and undergoes oxidative modification under the influence of vascular endothelial injury and oxidative stress, forming ox-LDL. After being taken up by macrophages in the vascular wall, ox-LDL forms foam cells, and the massive accumulation of foam cells forms lipid plaques, i.e., atherosclerotic plaques [106,107].

In contrast, HDL-C exerts a cholesterol reverse transport function, transporting cholesterol from peripheral tissues back to the liver for metabolism and excretion, thereby reducing cholesterol deposition in the vascular wall. When HDL-C levels decrease, the reverse transport function of HDL becomes weakened, leading to reduced cholesterol clearance from the vascular wall, further promoting lipid deposition and atherosclerotic plaque formation. In addition, elevated TG levels are closely associated with the occurrence of panvascular diseases. Hypertriglyceridemia potentially participates in the formation and progression of atherosclerosis through multiple pathways, such as affecting lipoprotein metabolism, promoting inflammatory responses, and inducing endothelial dysfunction [108,109].

#### 4.2.5. Platelet Aggregation and Thrombosis Are Key Factors in Infarction Events

Subendothelial collagen fibers are exposed after vascular endothelial injury. Platelets quickly recognize the injured site, adhere, and become activated. Activated platelets release various procoagulant factors and bioactive mediators, such as adenosine diphosphate (ADP) and thromboxane A2 (TXA2). These bioactive mediators further induce platelet aggregation, resulting in the formation of platelet emboli [110]. Furthermore, the procoagulant factors released by platelets activate the coagulation system, promoting the conversion of fibrinogen to fibrin. A fibrin network is then formed, which traps platelets, red blood cells, and other cellular components within it to form a thrombus [111,112].

The formation of a thrombus leads to vascular occlusion and ischemic tissue damage. For example, thrombus formation in the coronary arteries can cause acute myocardial infarction, whereas thrombus formation in the cerebral arteries can lead to stroke. In addition, after the rupture of unstable atherosclerotic plaques, the platelet and coagulation systems are rapidly activated, resulting in acute thrombus formation, which is the main mechanism underlying the occurrence of acute cardiovascular and cerebrovascular events [113,114,115].

**Figure 2:**
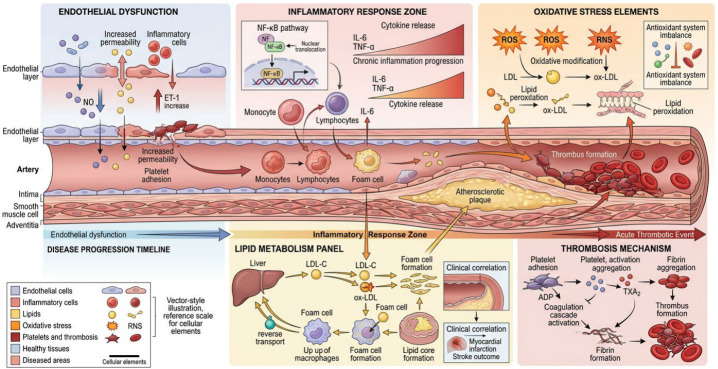
Multifactorial Pathogenesis of Panvascular Diseases: From Endothelial Dysfunction to Acute Thrombotic Events.

This schematic illustrates the interconnected molecular and cellular mechanisms driving panvascular disease progression, starting with endothelial dysfunction as the initiating event. Impaired endothelial integrity, characterized by reduced nitric oxide (NO) bioavailability, elevated endothelin-1 (ET-1), and increased vascular permeability, promotes inflammatory cell infiltration and platelet adhesion. This triggers the inflammatory response zone, where activation of the NF-κB pathway drives the release of pro-inflammatory cytokines (IL-6, TNF-α), recruiting monocytes and lymphocytes that differentiate into foam cells upon uptake of oxidized low-density lipoprotein (ox-LDL). Oxidative stress amplifies this cascade: reactive oxygen species (ROS) and reactive nitrogen species (RNS) mediate LDL oxidation, while an imbalanced antioxidant system exacerbates lipid peroxidation and endothelial damage. Dysregulated lipid metabolism—marked by increased hepatic LDL-C production and impaired reverse cholesterol transport—fuels foam cell formation and the development of atherosclerotic plaques. Ultimately, plaque rupture initiates the thrombosis mechanism, where platelet activation (via ADP, TXA_2_) and coagulation cascade activation lead to fibrin formation and thrombus formation, culminating in acute thrombotic events such as myocardial infarction and stroke. This timeline underscores the progressive nature of panvascular diseases, highlighting how endothelial dysfunction, inflammation, oxidative stress, lipid dysregulation, and thrombosis converge to drive pathological vascular remodeling and clinical sequelae (Figure 2).

## 5. The Active Compounds in *P. suffruticosa* Used to Prevent and Treat Panvascular Diseases

### 5.1. Active Compounds with Vascular Endothelial Cell Protective Effects

**Figure 3:**
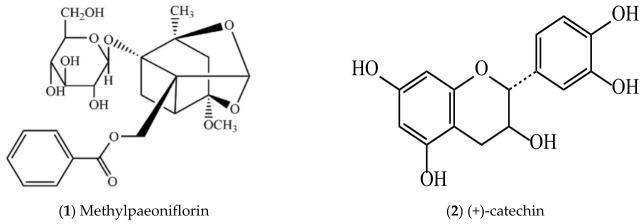

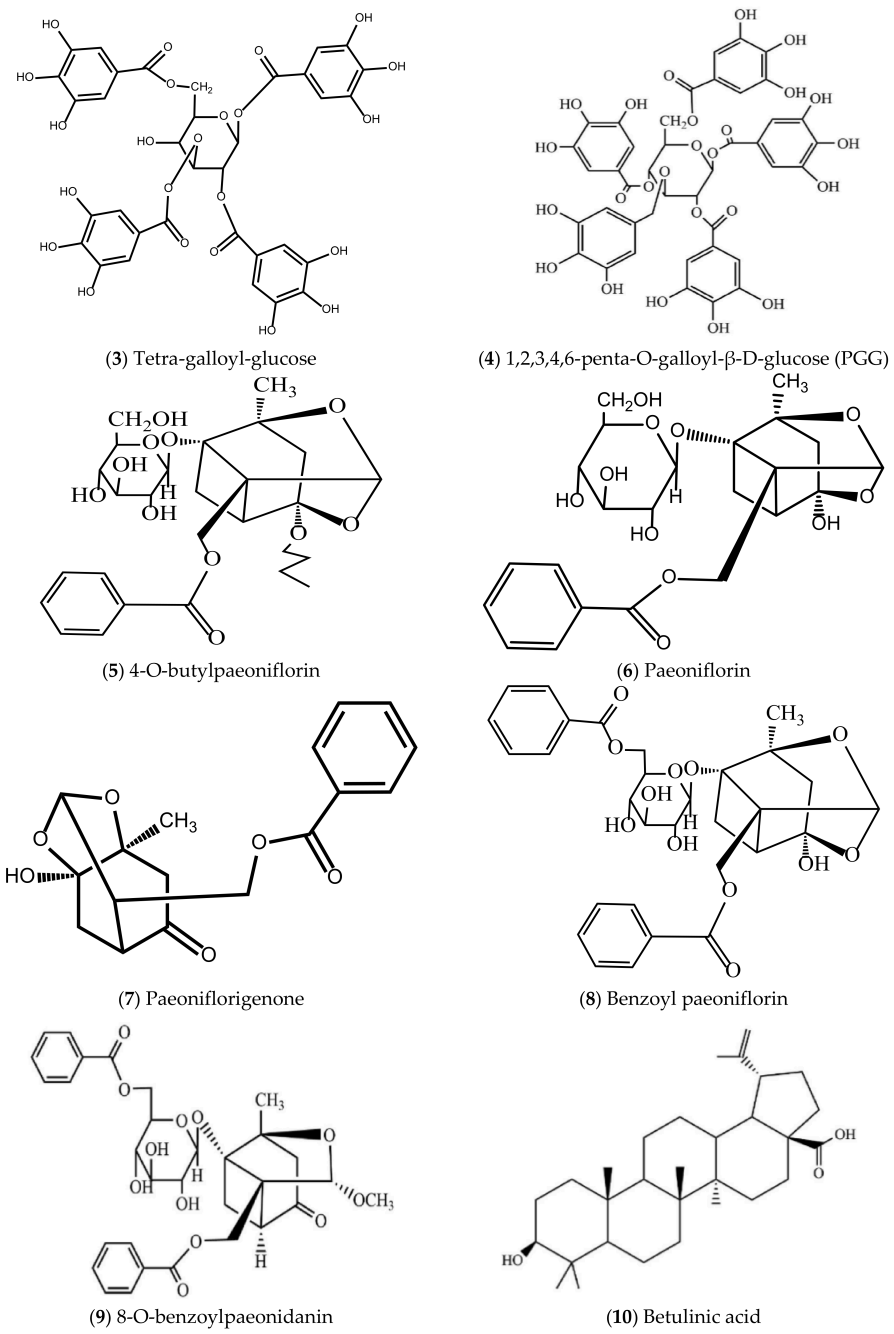

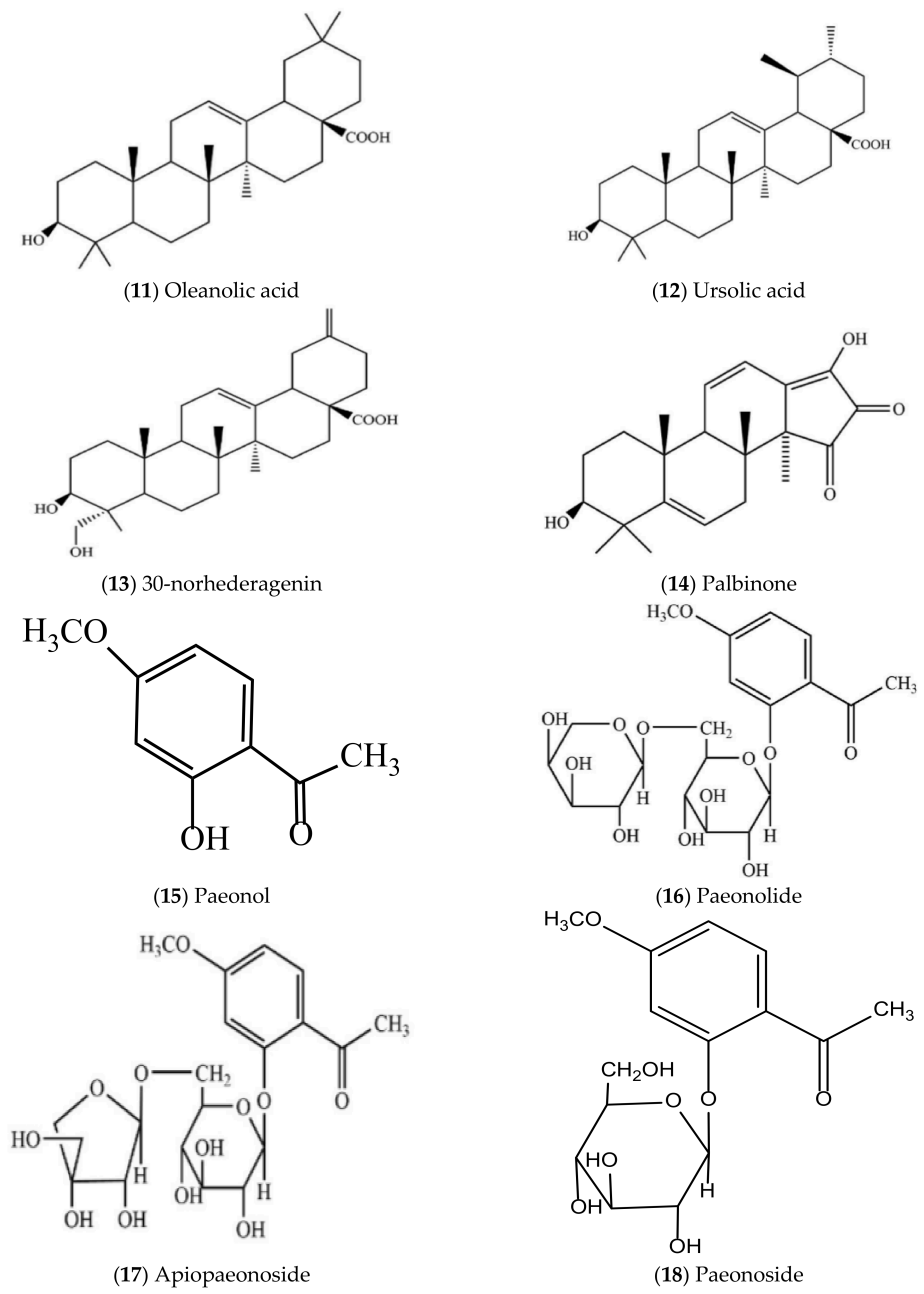

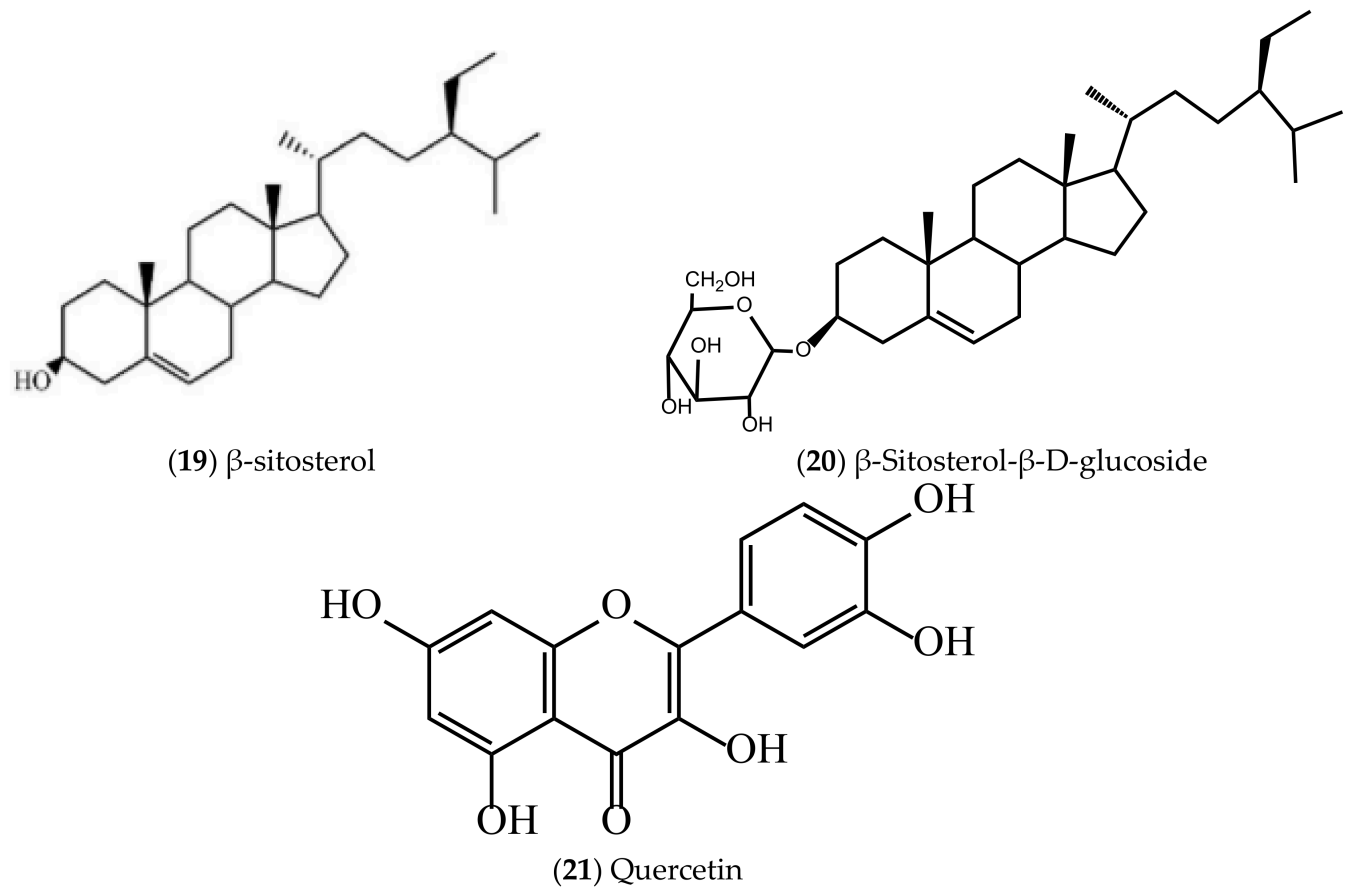
Structures of compounds with vascular endothelial cell protective effects in *P. suffruticosa*.

To systematically display the active compounds of *P. suffruticosa* with vascular endothelial protective effects and their distribution in plant parts for pharmaceutical use, Table 2 lists the names of the active compounds, their distribution in different plant parts for pharmaceutical use of *P. suffruticosa*, and the corresponding references. Meanwhile, Figure 3 shows the chemical structural formulas of these compounds, which intuitively reflects their structural characteristics and lays a foundation for the subsequent study of structure–activity relationships.

### 5.2. Active Compounds with Anti-Inflammatory Effects in P. suffruticosa

**Figure 4:**
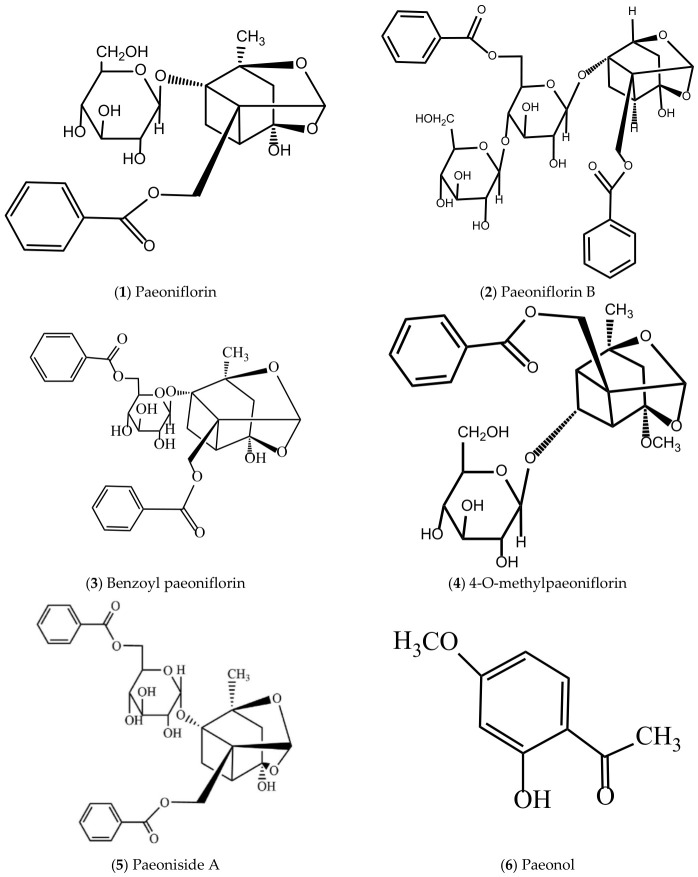

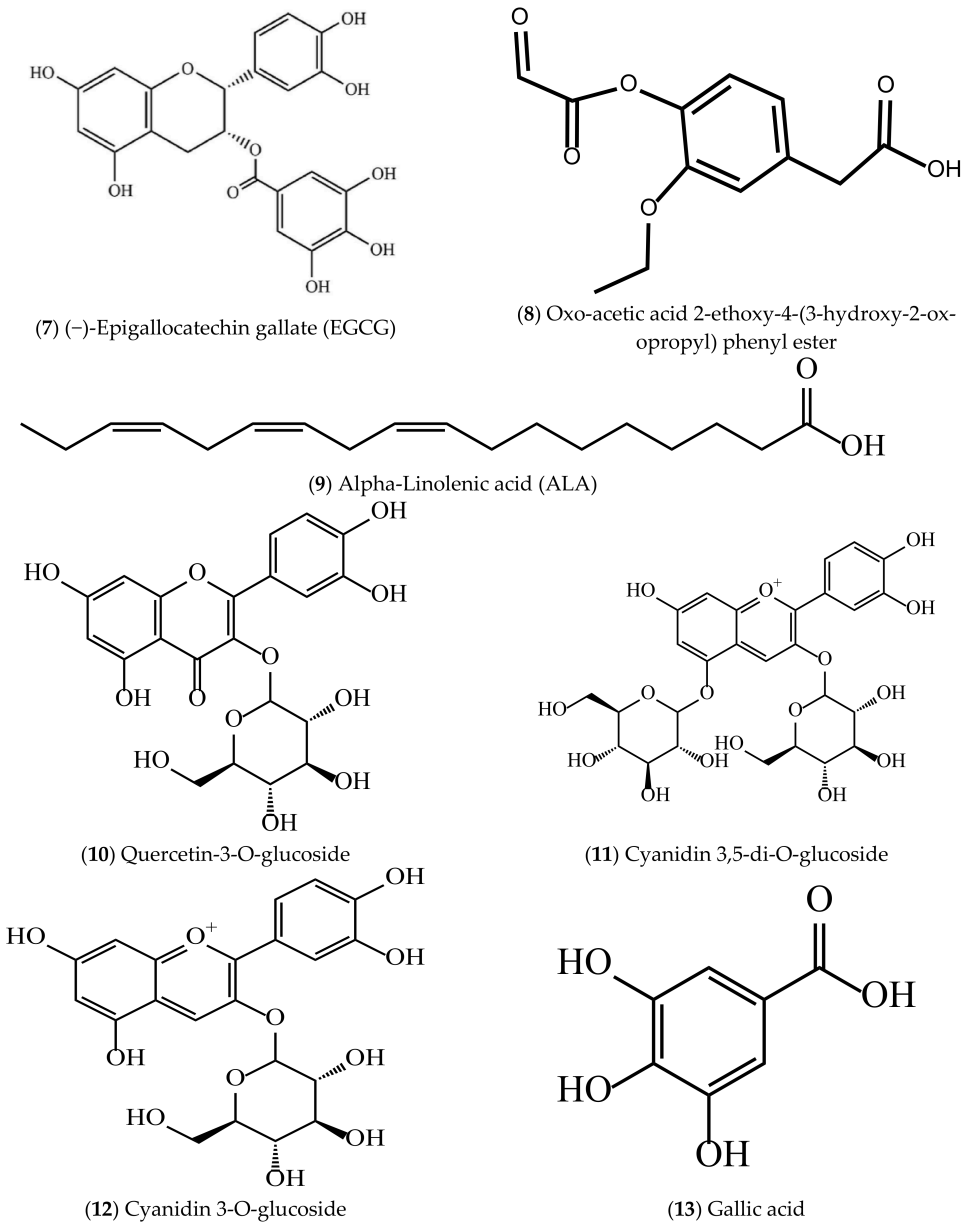

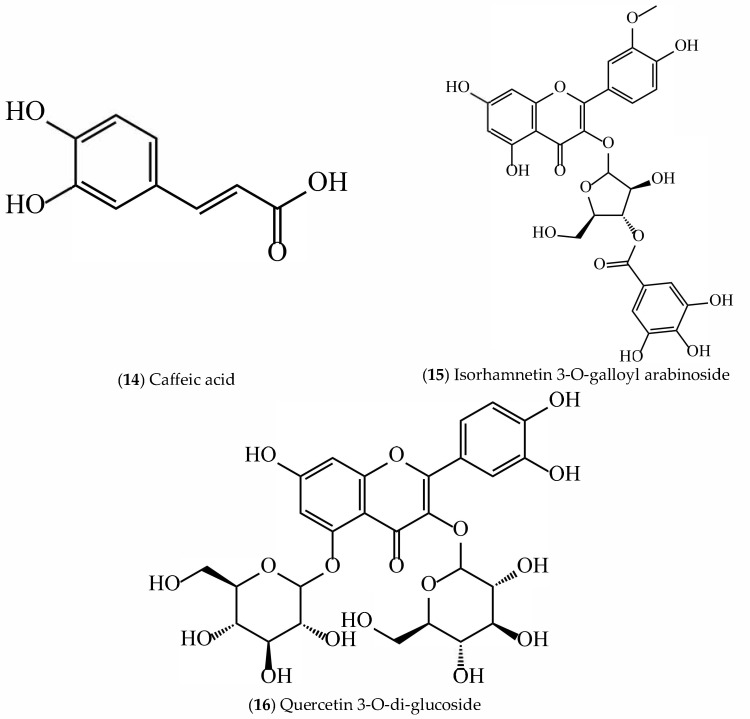
Structures of active compounds with anti-inflammatory effects in *P. suffruticosa*.

In order to clarify the anti-inflammatory active compounds derived from *P. suffruticosa* and their distribution characteristics, Table 3 summarizes the main anti-inflammatory compounds, their distribution in MC, leaves, petals, pollen, seeds and follicles, and relevant literature support. Figure 4 presents the chemical structures of these anti-inflammatory compounds, which helps to analyze their structure–activity relationship and action mechanism.

### 5.3. Active Compounds with Antioxidant Effects in P. suffruticosa

**Figure 5:**
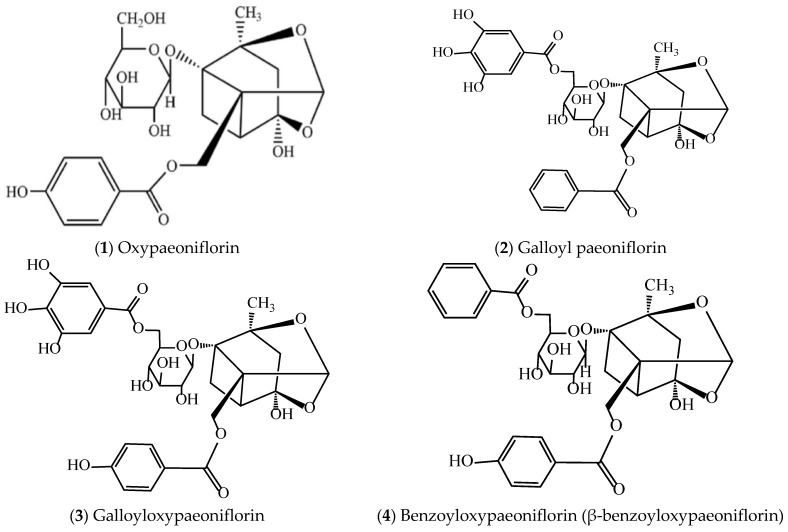

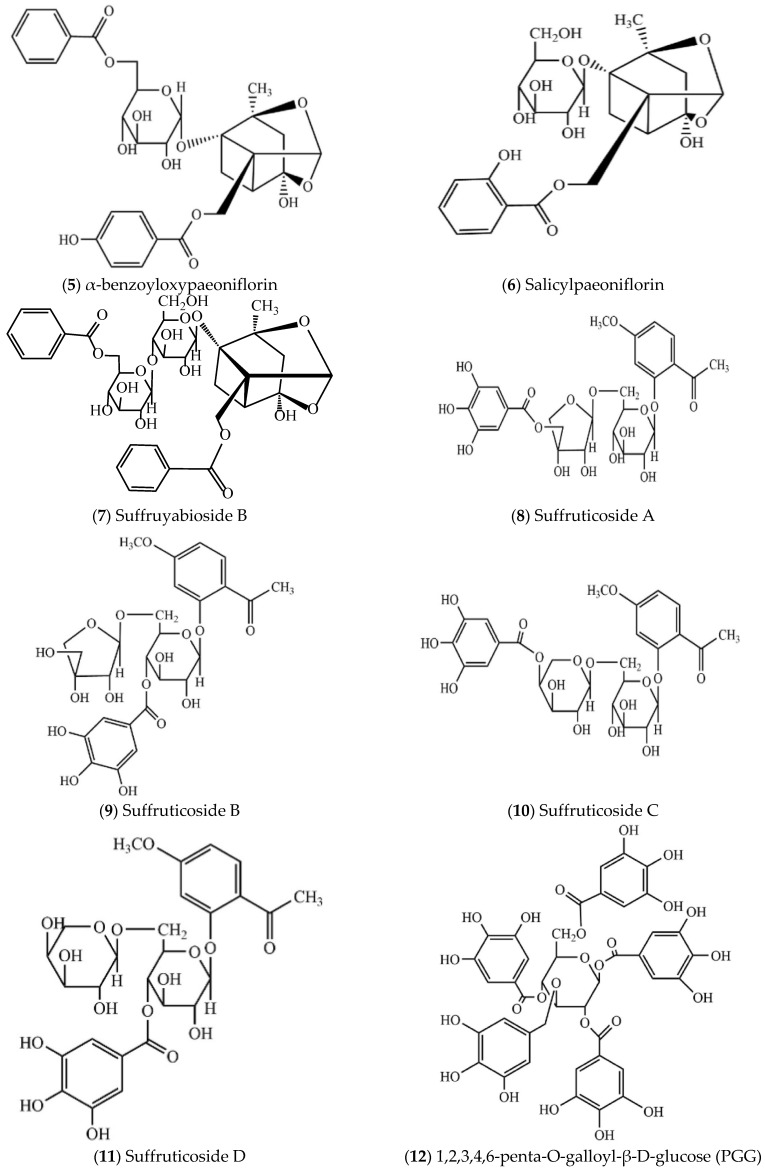

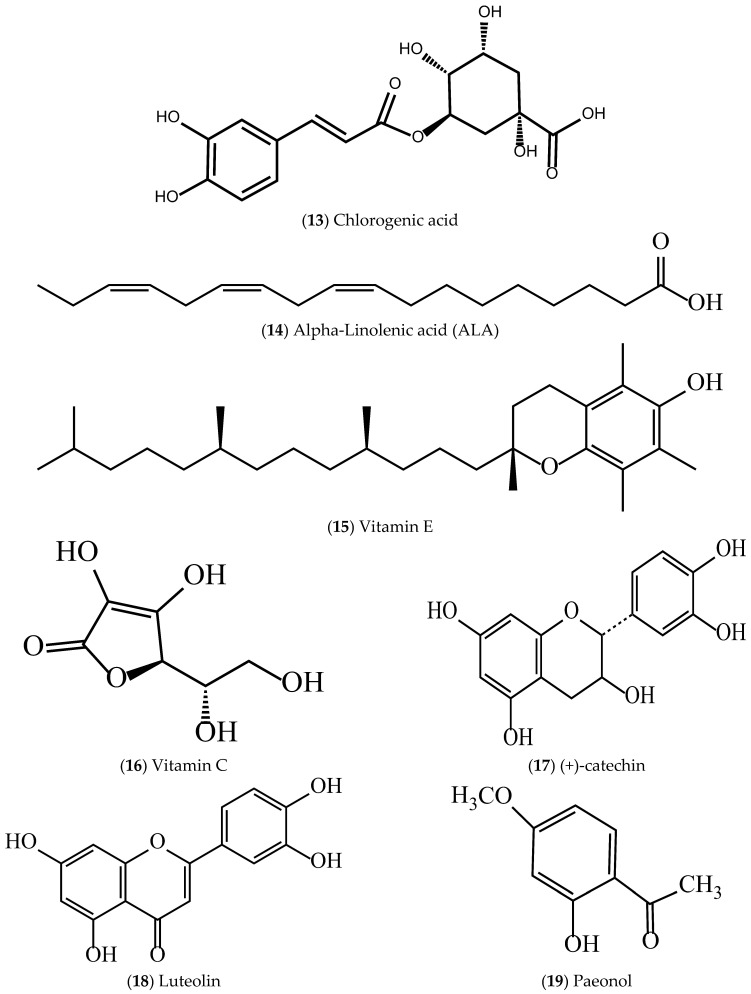

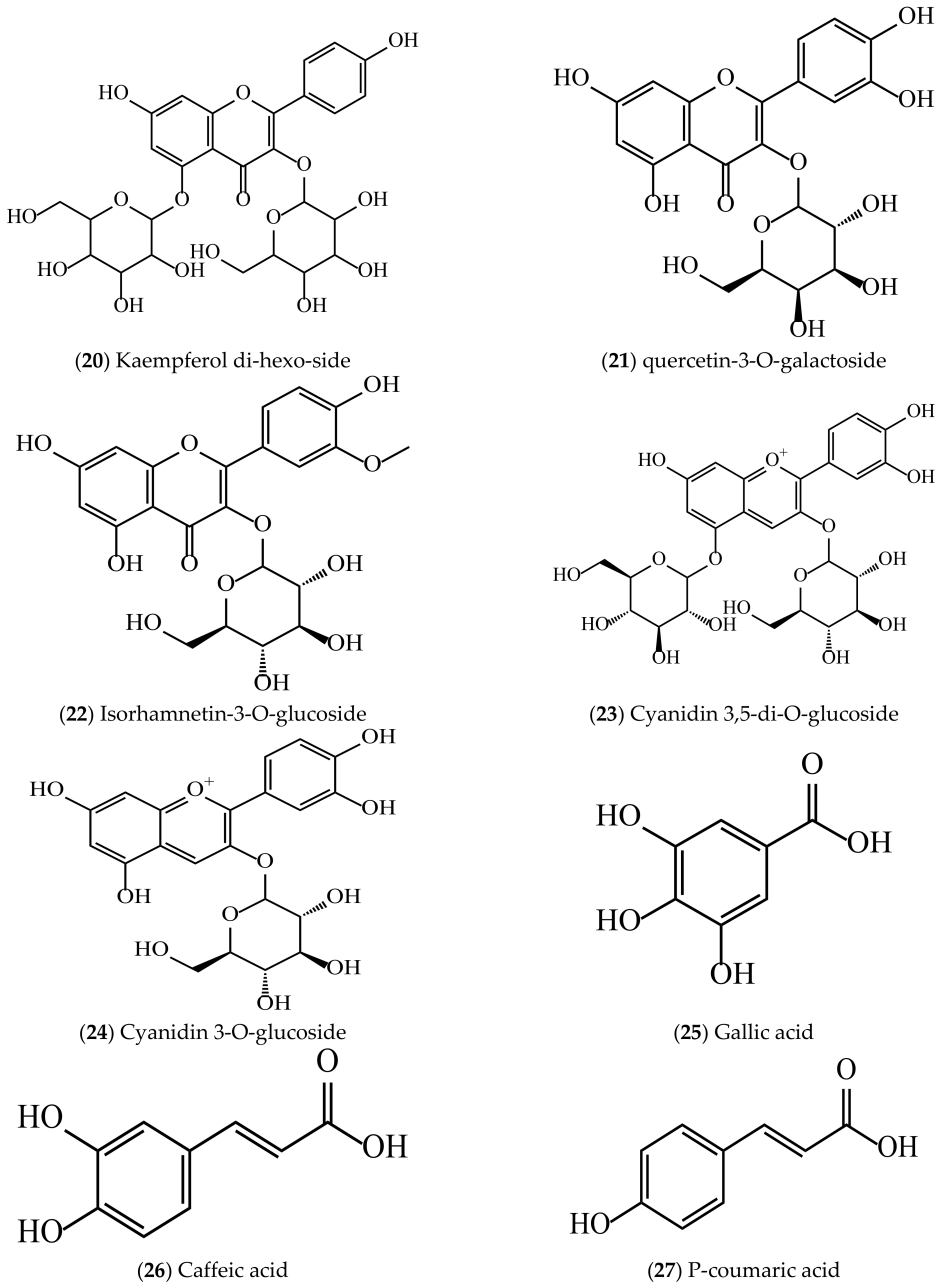

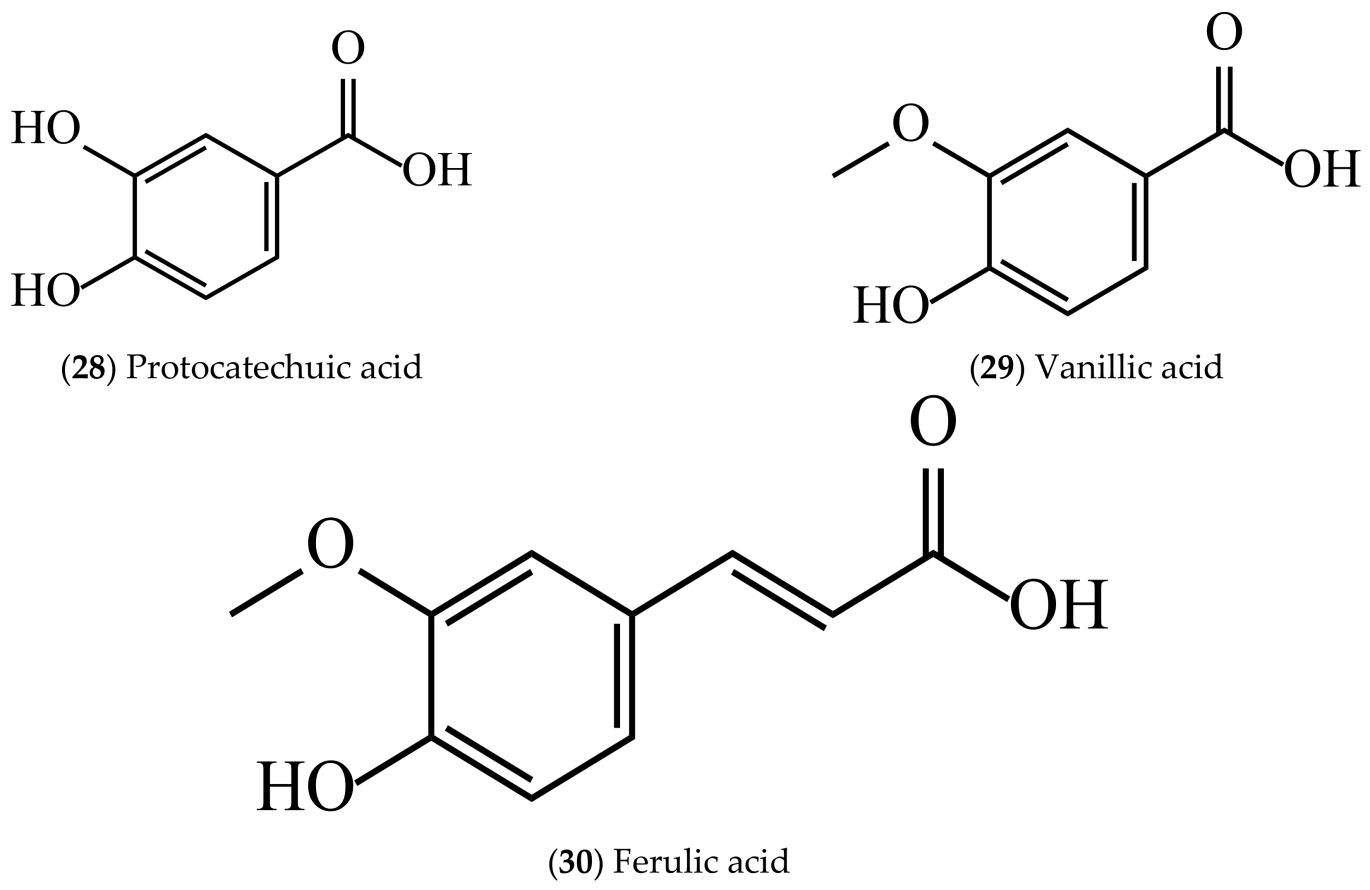
Structures of active compounds with antioxidant effects in *P. suffruticosa*.

Antioxidant active compounds are important material basis for *P. suffruticosa* to resist oxidative stress damage in vascular diseases. Table 4 collects the antioxidant compounds from different plant parts for pharmaceutical use of *P. suffruticosa*, their distribution and references. Figure 5 shows the chemical structures of these antioxidant compounds, which provides a structural basis for exploring their antioxidant mechanism and efficacy differences.

### 5.4. Active Compounds with Lipid Metabolism-Regulating Effects in P. suffruticosa

**Figure 6:**
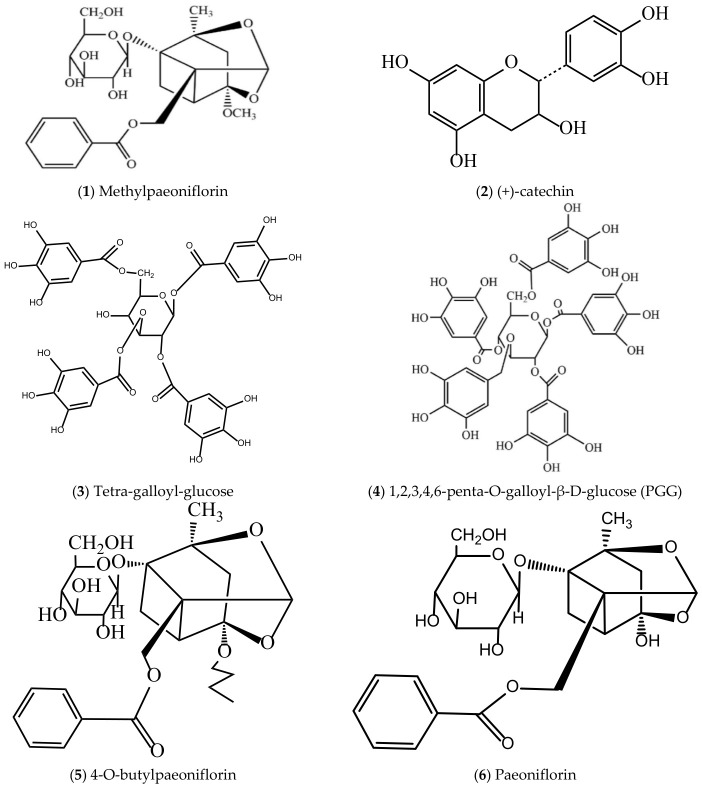

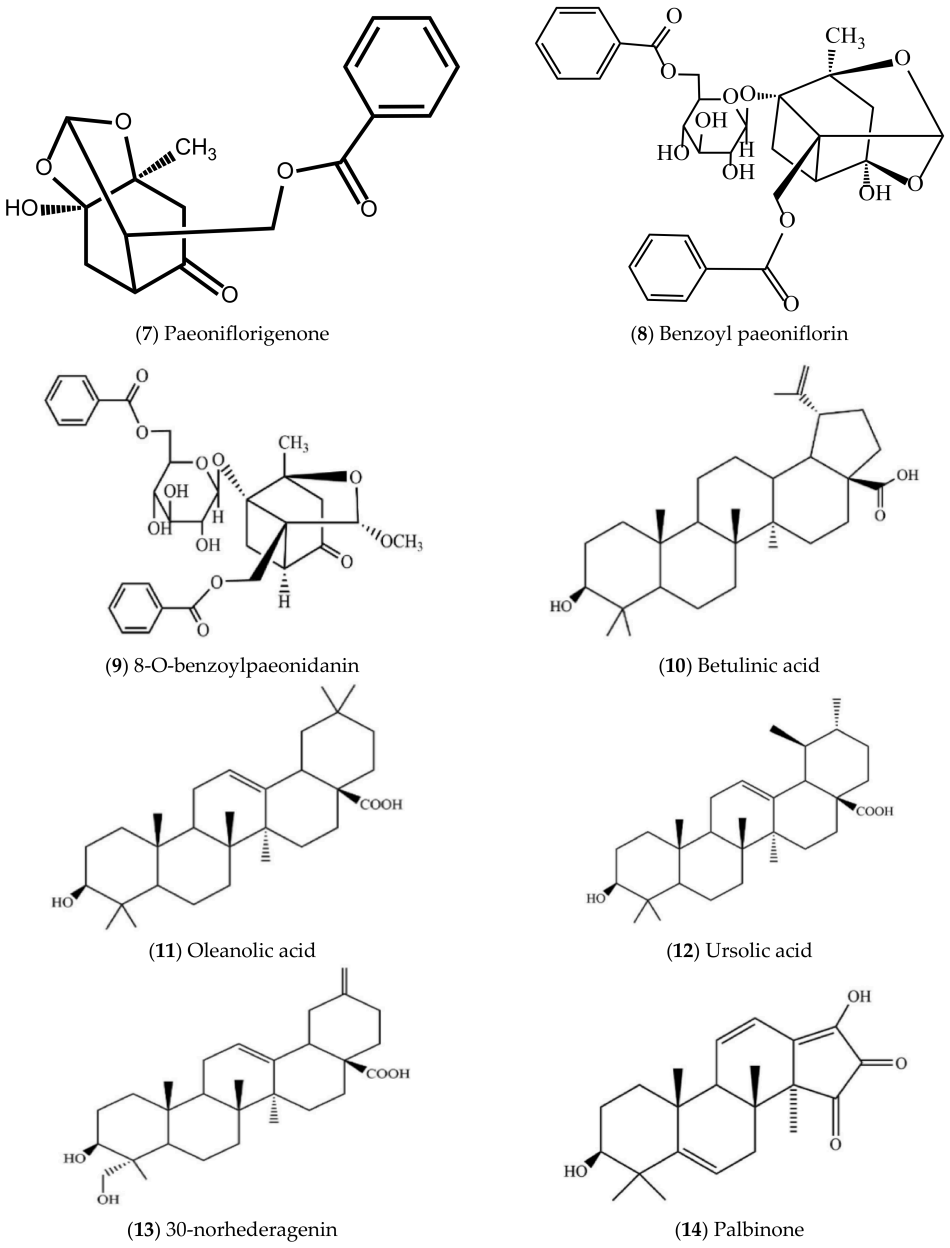

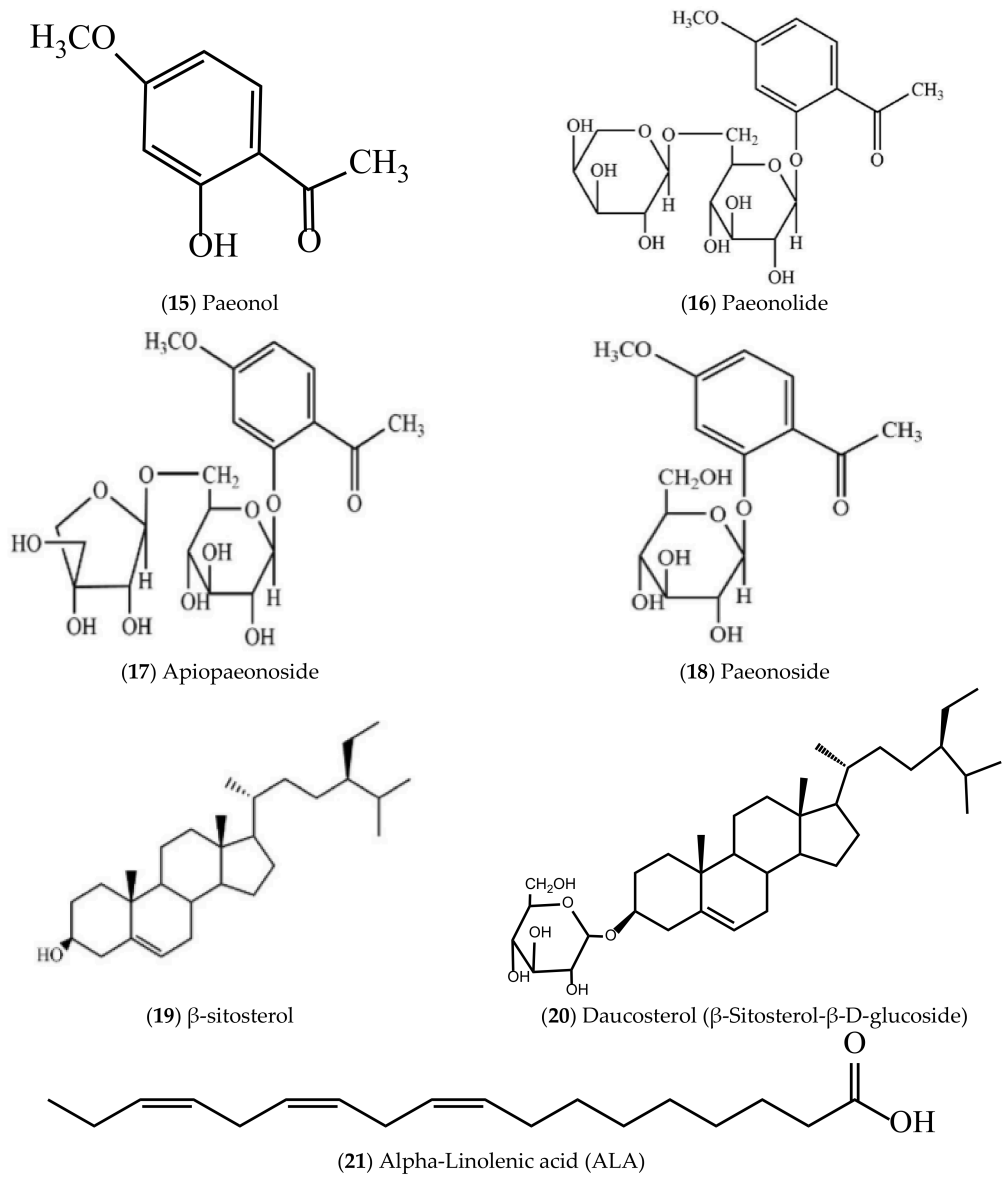
Structures of active compounds with lipid metabolism-regulating effects in *P. suffruticosa*.

To clarify the lipid-regulating active ingredients in *P. suffruticosa* and their distribution, Table 5 systematically summarizes the compounds with lipid metabolism regulation activity, their sources in different plant parts for pharmaceutical use and relevant references. Figure 6 shows the chemical structures of these lipid-regulating compounds, which is helpful to reveal the structural basis of their lipid-lowering and anti-atherosclerosis effects.

### 5.5. Active Compounds with Antiplatelet Aggregation and Antithrombosis Effects in P. suffruticosa

**Figure 7:**
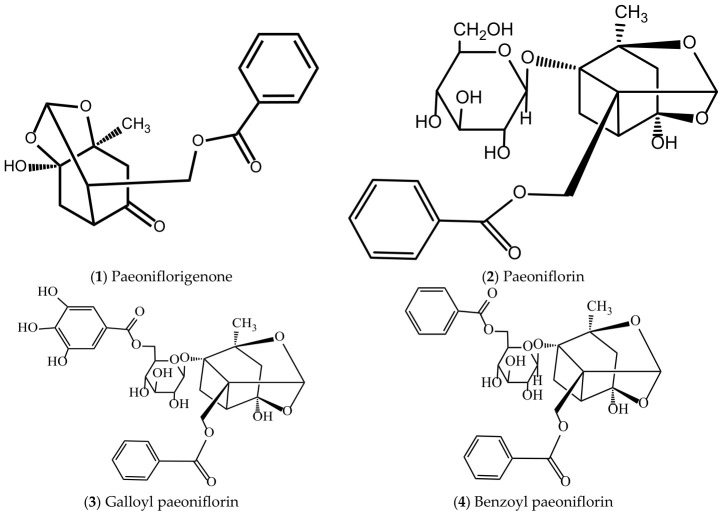

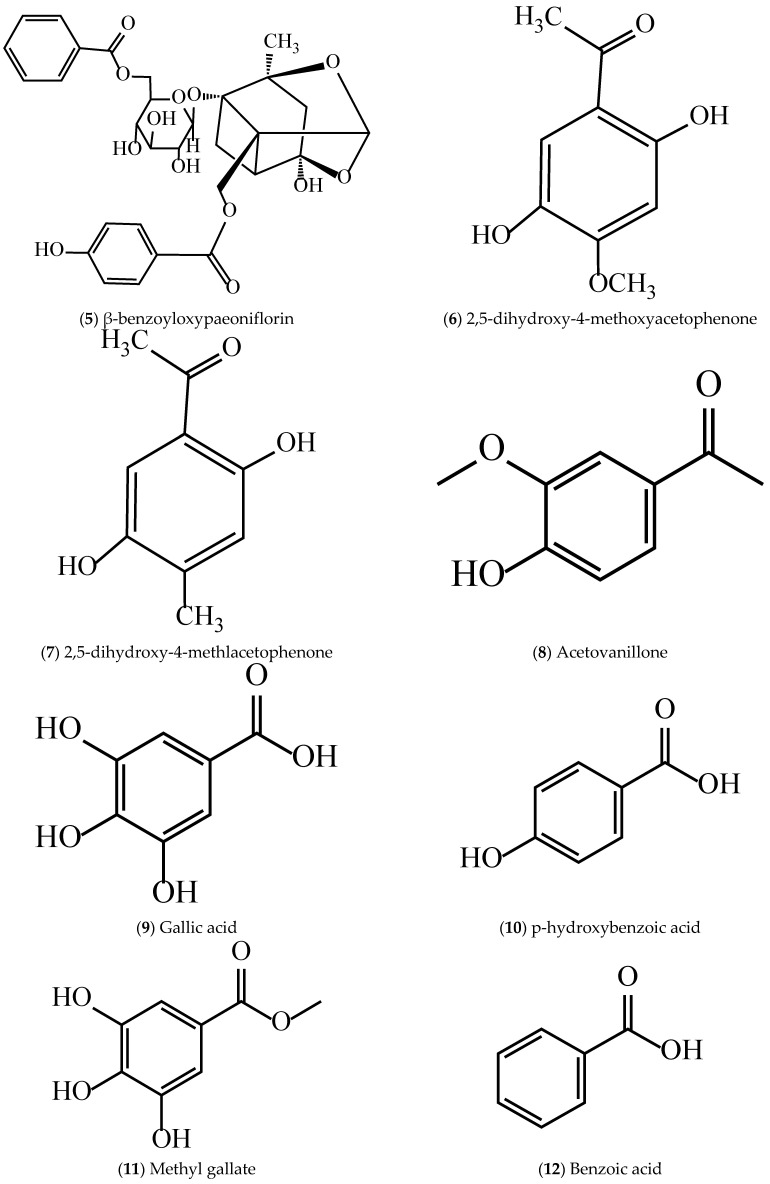
Structures of active compounds with antiplatelet aggregation and antithrombotic effects in *P. suffruticosa*.

Antiplatelet and antithrombotic compounds are crucial for preventing acute cardiovascular events. Table 6 summarizes the active compounds of *P. suffruticosa* that inhibit platelet aggregation and thrombosis, their distribution in different plant parts for pharmaceutical use and references. Figure 7 displays the chemical structures of these antithrombotic compounds, providing a structural basis for the development of antithrombotic drugs.

## 6. Prospects of Developing Innovative Drugs for Treating Panvascular Diseases Using *P. suffruticosa*’s Active Compounds

### 6.1. Basic Research: Deepening the Exploration of Mechanisms of Action and Structure–Activity Relationships

Most existing studies on the role of *P. suffruticosa* active compounds in preventing and treating panvascular diseases have focused on overall pharmacodynamic effects and partial signaling pathways. The underlying molecular mechanisms involved remain unclear. In the future, it is necessary to use techniques such as network pharmacology, molecular docking, and gene editing to systematically analyze the interactions between core compounds (e.g., paeonol, paeoniflorin) and target cells (e.g., vascular endothelial cells, macrophages, and smooth muscle cells) and clarify the precise regulatory mechanisms related to the key signaling pathways involved, including NF-κB, PI3K/Akt, and Nrf2. Meanwhile, regarding multi-component mixtures such as flavonoids and polysaccharides, isolation, purification, and activity tracing are required to identify the material basis and proportional relationship underlying their synergistic effects.

Additionally, research on structure–activity relationships is crucial for promoting the development of active compounds into effective drugs. For example, the phenolic hydroxyl structure of paeonol is closely related to its antioxidant and anti-inflammatory activities, while the glycoside structure of paeoniflorin may affect its bioavailability. Structural modification and transformation are needed to optimize the stability, targeting ability, and pharmacodynamic strength of active compounds, providing a basis for the development of high-efficiency and low-toxicity derivatives.

### 6.2. Resource Development: Promoting Whole-Plant Utilization and Improving Quality Standard Systems

All parts of *P. suffruticosa* contain active compounds; however, existing research has primarily focused on MC, with limited development and utilization of the leaves, petals, pollen, seeds, and follicles. In the future, based on the concept of whole-plant utilization, the value of non-traditional plant parts for pharmaceutical use should be systematically explored. This strategy can not only improve resource utilization efficiency, but also alleviate resource pressure caused by the over-reliance and excessive development of a single plant part.

Quality control is a prerequisite for the development of medicinal resources. Existing standards exist for the main components, such as MC and leaves, whereas quality standards for other components require improvement. It is necessary to combine modern analytical techniques (e.g., HPLC–MS and fingerprinting) to establish exclusive quality standards for different parts, clarify key indicators such as active compound content, heavy metal content, and pesticide residues, ensure the consistency and safety of raw materials, and provide guarantees for industrial production.

### 6.3. Research into Innovative Drugs: Simultaneously Advancing Diversified Dosage Forms and Compound Preparation Development

Innovative drug development exploiting the characteristics of *P. suffruticosa* active can be advanced in multiple directions as follows.

Monomer drug development: Monomers such as paeonol and paeoniflorin exhibit clear activities and can serve as lead compounds for the development of targeted drugs to prevent and treat atherosclerosis and thrombotic diseases. For example, paeonol can be formulated into sustained-release preparations to extend its action time, or nano-carrier technology can be utilized to enhance the vascular targeting of paeoniflorin.

Optimization of compound preparations: Drawing on the TCM theory of “monarch, minister, assistant and guide,” MC can be combined with other medicinal materials (e.g., *Salvia miltiorrhiza*, *Crataegus pinnatifida*) to develop compound preparations with anti-inflammatory, lipid-regulating, and antithrombotic effects, exerting multi-component synergistic effects to adapt to the complex pathological process of panvascular diseases.

Expansion of functional products: *P. suffruticosa* seed oil is rich in α-linolenic acid and can be developed into nutritional supplements for lipid regulation. Flavonoids in *P. suffruticosa* pollen can be utilized to create health foods that improve blood circulation, thereby realizing the diversified application of the “food and medicine homology” concept.

### 6.4. Clinical Application: Strengthening Evidence-Based Medicine and Exploring Combined Medication

Clinical translation is the ultimate goal of *P. suffruticosa* resource development. Currently, most studies remain at the cell and animal experiment stages, with insufficient clinical evidence. Thus, it is necessary to conduct multi-center, large-sample clinical trials to verify the efficacy and safety of *P. suffruticosa* active compounds in diseases such as coronary heart disease and cerebral infarction. Clarification of their applicable populations and dosage regimens is also required. Furthermore, the potential of combined application with existing drugs (e.g., statins, antiplatelet drugs) must be explored, including evaluations of synergistic effects and adverse reactions, providing a basis for optimizing clinical medication regimens.

### 6.5. Challenges and Outlook

Many challenges remain in the development of *P. suffruticosa* resources; for example, the isolation and purification processes of some compounds (e.g., polysaccharides and flavonoids) are complex and associated with high production costs, the in vivo metabolism processes and interactions of multiple compounds have not been fully clarified, and the depth and breadth of clinical research are insufficient. In the future, it will be necessary to achieve interdisciplinary cooperation to overcome technical bottlenecks and promote the in-depth integration of basic research and industrial applications.

In the future, *P. suffruticosa*, with its rich active compounds and unique pharmacological effects, is expected to become an important resource for the prevention and treatment of panvascular diseases. With in-depth research, its applications in innovative drugs, functional foods, and health products will continue to expand, providing diversified solutions for human health while promoting the modernization and internationalization of TCM resources.

Overall, the active compounds, pharmacological activities, and resource utilization of *P. suffruticosa* against panvascular diseases were systematically reviewed in this article to provide comprehensive insights. Furthermore, as a valuable medicinal and edible woody plant, the bioactive compounds and vascular-related pharmacological effects of *P. suffruticosa* deserve in-depth investigation to develop novel anti-panvascular disease agents and guide its rational clinical application and comprehensive resource development.

## Figures and Tables

**Table 1:** Specifications of the Major Plant Parts for Pharmaceutical Use of *P. suffruticosa*.

Specification	Identification/Preparation Process	Standard
Charred MC	Externally blackish-brown, internally brownish, texture loose and brittle	Tianjin [41]
MC	Thin slices, round, subround, or radially incised on one side	Shanghai [42]
Stir-fried MC	Cut surface pale yellow, slightly scorched aroma, scorched spots, few crystals visible	
Charred MC	Blackish-brown, fractured surface brownish, scorched aroma, crystals hardly visible	
MC	Thin slices, round or semicircular, texture hard and brittle, starchy nature	Jiangxi [43]
Stir-fried MC	Thin slices, round or semicircular, externally black, aroma and scorched spots	
MC	Thin round slices, brittle, starchy nature, cut surface pale pink	Henan [44]
Stir-fried MC	Thin round slices, slightly faint aroma, scorched spots	
Wine-processed MC	Thin round slices, darkened color, wine aroma	
Charred MC	Thin round slices, externally blackish-brown, internally charred brown	
Stir-fried MC	Thin slices, round or curled, shiny crystals sometimes visible on the inner surface	Sichuan [45]
MC	Thin slices, tubular or semi-tubular, shiny crystals commonly visible	Chongqing [46]
Stir-fried MC	Externally yellowish-brown, aromatic, taste slightly bitter and astringent	
MC	Thin slices, tubular or semi-tubular; outer surface grayish-brown or yellowish-brown, inner surface pale grayish-yellow or light brown	Guizhou [47]
MC	Thin subcircular slices, fine longitudinal textures, shiny crystals commonly visible	Shaanxi [48]
MC	Thin annular or semi-annular slices, surface white or pale pink	Jiangsu [49]
Stir-fried MC	Brownish-red, strong aroma, slight scorched spots	
Charred MC	Charred black, inner part brownish-yellow	
Stir-fried MC	Thin slices, round or curled, cut surface pale yellow, texture light and brittle	Zhejiang [50]
Charred MC	Thin slices, round or semicircular, externally blackish-brown, internally brown	Anhui [51]
MC	Thick slices, annular or semi-annular, shiny crystals commonly visible	Guangxi [52]
MC	Thin slices, nearly semi-arc-shaped, obvious fine longitudinal lines on the inner surface, shiny crystals commonly visible	Beijing [53]
MC	Thick slices, annular or semi-annular; outer surface grayish-brown or yellowish-brown, inner surface pale grayish-yellow or light brown, shiny crystals commonly visible	Hunan [54]
Charred MC	Thick slices, annular or semi-annular, externally blackish-brown, internally yellowish-brown	
Charred MC	Thin slices, round or semicircular, externally blackish-brown, internally brown	Shandong [55]
Charred MC	Round slices, outer surface charred black	Ningxia [56]
Charred MC	Thin slices, round or curled, externally blackish-brown, internally yellowish-brown or brown	Hubei [57]
Lian MC (taproots with intact bark)	Tubular or semi-tubular, with cracks, slightly curled inward or spread out	Chinese Pharmacopoeia [1]
Gua MC (peeled taproots)	Outer surface with scraper marks, reddish-brown or pale grayish-yellow, sometimes with grayish-brown spotted residual outer bark	
MC Decoction Pieces	Thin slices, round or curled; outer surface of Lian Moutan Cortex grayish-brown or yellowish-brown, outer surface of Gua Moutan Cortex reddish-brown or pale grayish-yellow	
MC (Charred Decoction Pieces)	Surface charred brown, brittle and easy to break	Journal [58]
*P. suffruticosa* Leaves	In bundles, leaves mostly shrunken and curled	Shandong [55]
*P. suffruticosa* Leaves	Harvested in autumn, dried, crushed and passed through a No. 4 sieve	Journal [8]
*P. suffruticosa* Leaves	Dried leaves, extracted with 70% methanol under ultrasonic conditions for 45 min	Journal [59]
*P. suffruticosa* Petals	Freeze-dried followed by ultra-fine pulverization, cell wall breaking rate of 100%	Journal [60]
*P. suffruticosa* Petals	Shade-dried or oven-dried, crushed and passed through a No. 4 sieve	Journal [61]
*P. suffruticosa* Pollen	Wall-broken pollen, mechanical wall breaking (wall breaking rate: 89.16%)	Journal [62]
*P. suffruticosa* Pollen (Pollen Buccal Tablets)	Wall-broken pollen mixed with excipients, prepared by wet granulation and tableting	Journal [25]
*P. suffruticosa* Seeds	No special processing, graded by quality	Journal [63]
*P. suffruticosa* Seeds	Extracted by pressing method, aqueous enzymatic method, or leaching method	Journal [64]
*P. suffruticosa* Follicles	Crushed and passed through a No. 4 sieve; microscopic identification shows cluster crystals and vessels	Journal [28]

**Table 2:** Active Compounds of *P. suffruticosa* with Vascular Endothelial Cell Protective Effects.

Serial Number	Name	*P. suffruticosa* Component	References
**1**	Methylpaeoniflorin (3-O-methylpaeoniflorin)	MC, petals, pollen, seeds	[116]
**2**	(+)-catechin	MC, leaves, petals, pollen	[13]
**3**	Tetra-galloyl-glucose	MC	[116]
**4**	1,2,3,4,6-penta-O-galloyl-β-D-glucose (PGG)	MC, petals, pollen	[116]
**5**	4-O-butylpaeoniflorin	MC	[117]
**6**	Paeoniflorin	MC, leaves, petals, pollen, seeds, follicles	[13,118]
**7**	Paeoniflorigenone	MC, petals, pollen, follicles	[13,119]
**8**	Benzoyl paeoniflorin	MC, leaves, petals, follicles	[13,119]
**9**	8-O-benzoylpaeonidanin	MC	[117]
**10**	Betulinic acid	MC	[120]
**11**	Oleanolic acid	MC, petals, seeds, follicles	[120,121]
**12**	Ursolic acid	MC, petals, seeds	[120]
**13**	30-norhederagenin	MC	[117]
**14**	Palbinone	MC	[117,120]
**15**	Paeonol	MC, leaves, petals, pollen, follicles	[119,121]
**16**	Paeonolide	MC, petals, pollen	[119]
**17**	Apiopaeonoside	MC	[119]
**18**	Paeonoside	MC, petals, pollen, follicles	[119]
**19**	β-sitosterol	MC, petals, pollen, seeds, follicles	[120,121]
**20**	Daucosterol (β-Sitosterol-β-D-glucoside)	MC, petals, pollen, follicles	[120,121]
**21**	Quercetin	MC, petals, pollen	[122,123]

**Table 3:** Active Compounds with Anti-Inflammatory Effects in *P. suffruticosa*.

Serial Number	Name	*P. suffruticosa* Component	References
**1**	Paeoniflorin	MC, leaves, petals, pollen, seeds, follicles	[124]
**2**	Paeoniflorin B	MC	[125]
**3**	Benzoyl paeoniflorin	MC, leaves, petals, pollen, follicles	[124]
**4**	4-O-methylpaeoniflorin	MC	[125]
**5**	Paeoniside A	MC	[124]
**6**	Paeonol	MC, leaves, petals, pollen, follicles	[119,121]
**7**	(−)-Epigallocatechin gallate (EGCG)	MC, leaves	[126]
**8**	Oxo-acetic acid 2-ethoxy-4-(3-hydroxy-2-oxopropyl) phenyl ester	MC	[127]
**9**	Alpha-Linolenic acid (ALA)	Seeds	[128,129]
**10**	Quercetin-3-O-glucoside	MC, leaves, petals, pollen	[130]
**11**	Cyanidin 3,5-di-O-glucoside	Petals, pollen	[130]
**12**	Cyanidin 3-O-glucoside	Petals, pollen	[130]
**13**	Gallic acid	MC, leaves, follicles	[121,131]
**14**	Caffeic acid	MC, leaves, follicles	[121,132]
**15**	Isorhamnetin 3-O-galloyl arabinoside	Petals, pollen	[130]
**16**	Quercetin 3-O-di-glucoside	Petals, pollen	[130]

**Table 4:** Active Compounds with Antioxidant Effects in *P. suffruticosa*.

Serial Number	Name	*P. suffruticosa* Component	References
**1**	Oxypaeoniflorin	MC, petals, pollen	[133]
**2**	Galloyl paeoniflorin	MC, petals, pollen	[125]
**3**	Galloyloxypaeoniflorin	MC, petals, pollen	[134]
**4**	Benzoyloxypaeoniflorin (β-benzoyloxypaeoniflorin)	MC, petals, pollen	[135]
**5**	α-benzoyloxypaeoniflorin	MC, petals, pollen	[136]
**6**	Salicylpaeoniflorin	MC, petals, pollen	[137]
**7**	Suffruyabioside B	MC, petals, pollen	[118]
**8**	Suffruticoside A	MC	[138]
**9**	Suffruticoside B	MC	[133,138]
**10**	Suffruticoside C	MC	[133]
**11**	Suffruticoside D	MC	[133]
**12**	1,2,3,4,6-penta-O-galloyl-β-D-glucose (PGG)	MC, petals, pollen	[116]
**13**	Chlorogenic acid	MC, leaves, petals, pollen, follicles	[121,139]
**14**	Alpha-Linolenic acid (ALA)	Seeds	[140]
**15**	Vitamin E	MC, leaves, petals, pollen, seeds, follicles	[121,141]
**16**	Vitamin C	MC, leaves, petals, pollen, follicles	[121,141]
**17**	(+)-catechin	MC, petals, pollen	[142]
**18**	Luteolin	MC, petals, pollen	[142]
**19**	Paeonol	MC, leaves, petals, pollen, follicles	[121,142]
**20**	Kaempferol di-hexo-side	MC, petals, pollen	[143]
**21**	Quercetin-3-O-galactoside	MC, petals, pollen	[143]
**22**	Isorhamnetin-3-O-glucoside	MC, petals, pollen	[143]
**23**	Cyanidin 3,5-di-O-glucoside	Petals, pollen	[130]
**24**	Cyanidin 3-O-glucoside	Petals, pollen	[130]
**25**	Gallic acid	MC, leaves, petals, pollen	[144]
**26**	Caffeic acid	MC, leaves, petals, pollen, follicles	[121,144]
**27**	P-coumaric acid	MC, petals, pollen	[144]
**28**	Protocatechuic acid	MC, petals, pollen	[144]
**29**	Vanillic acid	MC, petals, pollen	[144]
**30**	Ferulic acid	MC, petals, pollen	[144]

**Table 5:** Active Compounds with Lipid Metabolism-Regulating Effects in *P. suffruticosa*.

Serial Number	Name	*P. suffruticosa* Component	References
**1**	Methylpaeoniflorin (3-O-methylpaeoniflorin)	MC, petals, pollen, seeds	[116]
**2**	(+)-catechin	MC, leaves, petals, pollen	[13]
**3**	Tetra-galloyl-glucose	MC	[116]
**4**	1,2,3,4,6-penta-O-galloyl-β-D-glucose (PGG)	MC, petals, pollen	[116]
**5**	4-O-butylpaeoniflorin	MC	[117]
**6**	Paeoniflorin	MC, leaves, petals, pollen, seeds, follicles	[13,118]
**7**	Paeoniflorigenone	MC, petals, pollen, follicles	[13,119]
**8**	Benzoyl paeoniflorin	MC, leaves, petals, pollen, follicles	[13,119]
**9**	8-O-benzoylpaeonidanin	MC	[117]
**10**	Betulinic acid	MC	[120]
**11**	Oleanolic acid	MC, petals, pollen, seeds, follicles	[120,121]
**12**	Ursolic acid	MC, petals, pollen, seeds	[120]
**13**	30-norhederagenin	MC	[117]
**14**	Palbinone	MC	[117,120]
**15**	Paeonol	MC, leaves, petals, pollen, follicles	[119,121]
**16**	Paeonolide	MC, petals, pollen	[119]
**17**	Apiopaeonoside	MC	[119]
**18**	Paeonoside	MC, petals, pollen, follicles	[119]
**19**	β-sitosterol	MC, petals, pollen, seeds, follicles	[120,121]
**20**	Daucosterol (β-Sitosterol-β-D-glucoside)	MC, petals, pollen, follicles	[120,121]
**21**	Alpha-Linolenic acid (ALA)	Seeds	[145]

**Table 6:** Active Compounds with Antiplatelet Aggregation and Antithrombosis Effects in *P. suffruticosa*.

Serial Number	Name	*P. suffruticosa* Component	References
**1**	Paeoniflorigenone	MC, petals, pollen, follicles	[119,121]
**2**	Paeoniflorin	MC, leaves, petals, pollen, follicles	[119,121]
**3**	Galloyl paeoniflorin	MC, petals, pollen, follicles	[121,133]
**4**	Benzoyl paeoniflorin	MC, leaves, petals, pollen, follicles	[13,121]
**5**	Benzoyloxypaeoniflorin (β-benzoyloxypaeoniflorin)	MC, petals, pollen	[13]
**6**	2,5-dihydroxy-4-methoxyacetophenone	MC, leaves, petals, pollen	[146]
**7**	2,5-dihydroxy-4-methlacetophenone	MC, leaves, petals, pollen	[146]
**8**	Acetovanillone	MC, leaves, petals, pollen	[147]
**9**	Gallic acid	MC, leaves, petals, pollen, follicles	[121,148]
**10**	p-hydroxybenzoic acid	MC, leaves, petals, pollen, follicles	[121,148]
**11**	Methyl gallate	MC, leaves, petals, pollen	[13]
**12**	Benzoic acid	MC, leaves, petals, pollen, seeds, follicles	[121,149]

## Data Availability

Data will be made available on request.

## Abbreviations

*P. suffruticosa*, *Paeonia* × *suffruticosa* Andrews; MC, Moutan Cortex; NO, nitric oxide; PAF, platelet-activating factor; NETs, neutrophil extracellular traps; LDL, low-density lipoprotein; LDL-C, low-density lipoprotein cholesterol; oxLDL, oxidized LDL; MCP-1, monocyte chemotactic protein-1; ICAM-1, intracellular adhesion molecule-1; VCAM-1, vascular cell adhesion molecule; ROS, reactive oxygen species; LPS, Lipopolysaccharide; ET-1, Endothelin-1; H_2_O_2_, hydrogen peroxide; ONOO-, peroxynitrite; HOCl, hypochlorous acid; O2·^−^, superoxide; HO·, hydroxyl species; eNOS, endothelial nitric oxide synthase; iNOS, inducible nitric oxide synthase; NOX1, NOX2 and NOX5, NADPH oxidases; BH4, tetrahydrobiopterin; SOD, superoxide dismutase; GPX, glutathione peroxidase; TRX, thioredoxin; PON, Paraoxonase; HDL, high density lipoprotein; HDL-C, high-density lipoprotein cholesterol; NF-κB, nuclear factor-kappa B; TC, total cholesterol; TG, triglycerides; IL-1β, interleukin-1β; TxA2, thromboxane A2; ADP, adenosine diphosphate; IL-6, interleukin6; VSMC, vascular smooth muscle cell; PGG, 1,2,3,4,6-penta-O-galloyl-β-D-glucose; ALA, Alpha-Linolenic acid; EGCG, (−)-Epigallocatechin gallate; Eos, Essential oils; GC-MS, Gas Chromatography-Mass Spectrometry.

## References

[1] National Pharmacopoeia Commission (2025). Pharmacopoeia of the People’s Republic of China.

[2] Peng H., Wang D., Peng D., Huang L. (2017). Research and investigation on original plants of medicinal Moutan. China J. Chin. Mater. Medica.

[3] Ma L., Jiao K., Luo L., Xiang J., Fan J., Zhang X., Yi J., Zhu W. (2019). Characterization and macrophage immunomodulatory activity of two polysaccharides from the flowers of *Paeonia suffruticosa* Andr. Int. J. Biol. Macromol..

[4] Tang J., Zhou G., Lu Y., Shi S., Cheng L., Xiang J., Wan S., Wang M. (2024). Panvascular concept in the evaluation and treatment of intracranial atherosclerotic stenosis. Front. Neurol..

[5] Chan A.W. (2004). Expanding roles of the cardiovascular specialists in panvascular disease prevention and treatment. Can. J. Cardiol..

[6] Sun Z., Du J., Hwang E., Yi T. (2018). Paeonol extracted from *Paeonia suffruticosa* Andr. ameliorated UVB-induced skin photoaging via DLD/Nrf2/ARE and MAPK/AP-1 pathway. Phytother. Res..

[7] Gao Z., Zhang Y., Ren L., Wang X., Bai M., Gao Y., Zhang Y. (2021). Research Progress and Application of *P. suffruticosa* Leaves. Yunnan Chem. Technol..

[8] Xiao H., Zhang Y., Yang X., Li J., Lin F., Luo D., Wang S. (2021). Study on Quality Standard of *P. suffruticosa* Leaves. Northwest Pharm. J..

[9] Huo Y. (2024). Study on Bioactivity and Secondary Metabolites of P. suffruticosa Petals.

[10] Shao Y., Qiao Q. (2025). Research Progress on Active Compounds and Efficacy of *P. suffruticosa* Flowers. North. Hortic..

[11] Bertoni C., Abodi M., D’Oria V., Milani G.P., Agostoni C., Mazzocchi A. (2023). Alpha-linolenic acid and cardiovascular events: A narrative review. Int. J. Mol. Sci..

[12] Xiao H., Yang X., Zhao L., Li C., Lin F., Wang S., Gao J. (2024). Study on Quality Standard of *P. suffruticosa* Follicles. Northwest Pharm. J..

[13] Koo Y.K., Kim J.M., Koo J.Y., Kang S.S., Bae K., Kim Y.S., Chung J.H., Yun-Choi H.S. (2010). Platelet anti-aggregatory and blood anti-coagulant effects of compounds isolated from *Paeonia lactiflora* and *Paeonia suffruticosa*. Die Pharm. Int. J. Pharm. Sci..

[14] Gu Y., Chen K., Xi B., Xie J., Bing X. (2022). Paeonol increases the antioxidant and anti-inflammatory capacity of gibel carp (*Carassius auratus* gibelio) challenged with *Aeromonas hydrophila*. Fish Shellfish Immunol..

[15] Ma X., Zhang W., Jiang Y., Wen J., Wei S., Zhao Y. (2020). Paeoniflorin, a natural product with multiple targets in liver diseases—A mini review. Front. Pharmacol..

[16] Zhang L.X., Chang Q.S., He Y.L., Zhao X.L., Liu W., Guo Q., Chen K., Hou X.G. (2024). Selenite foliar application increased the accumulation of medicinal components in *Paeonia ostii* by promoting antioxidant capacity, reducing oxidative stress, and improving photosynthetic capacity. Photosynthetica.

[17] Qian W., Li X., Yang M., Mao G. (2021). Antibacterial and anti-biofilm activities of paeonol against *Klebsiella pneumoniae* and *Enterobacter cloacae*. Biofouling.

[18] Wang J., Wu G., Chu H., Wu Z., Sun J. (2020). Paeonol derivatives and pharmacological activities: A review of recent progress. Mini Rev. Med. Chem..

[19] Ni J., Yang M., Zheng X., Wang M., Xiao Q., Han H., Dong P. (2024). Synthesis, antioxidant activity, and molecular docking of novel paeoniflorin derivatives. Chem. Biol. Drug Des..

[20] Xie H., Xie Z., Luan F., Zeng J., Zhang X., Chen L., Zeng N., Liu R. (2024). Potential therapeutic effects of Chinese herbal medicine in postpartum depression: Mechanisms and future directions. J. Ethnopharmacol..

[21] Yan X., Liu H., Zou Y., Ren Z. (2008). Physiological activities and research advance in synthesis of flavonoids. Chin. J. Org. Chem..

[22] Li N., Yu X., Yu Q., Wang M. (2019). Research progress on stability of polysaccharides in traditional Chinese medicine. Zhongguo Zhong Yao Za Zhi = Zhongguo Zhongyao Zazhi = China J. Chin. Mater. Medica.

[23] Du J., Bai B., Yu Y., Wang C., Qian Z. (2005). The new progress of the study about volatile oil of the angelica. Zhongguo Zhong Yao Za Zhi = Zhongguo Zhongyao Zazhi = China J. Chin. Mater. Medica.

[24] Hao Y., Ji H., Gao L., Chen J., Wang X., Hao H., Li J. (2025). Determination of Compound Content, Functional Characteristics and Application of *P. suffruticosa* Pollen. China Food Addit..

[25] Li Z., Li L., Shi X., Fan B. (2019). Study on Quality Standard of *P. suffruticosa* Pollen Buccal Tablets. China Pharm..

[26] Wei Y., Huang Z., Zhao L., Yuan W. (2023). Research Progress on Extraction Technology and Bioactivity of *P. suffruticosa* Seed Oil. Cereals Oils.

[27] Bertoni C., Pini C., Mazzocchi A., Agostoni C., Brambilla P. (2024). The role of alpha-linolenic acid and other polyunsaturated fatty acids in mental health: A narrative review. Int. J. Mol. Sci..

[28] Wei X., Wang F., Wang X., Yi X., He H., Liu X., Yang Y., Kerboua I. (2024). The one-step synthesis of Fe-N co-doped peony pod-based porous carbon for the removal of tetracycline. Ind. Crop. Prod..

[29] Qin X., Xu L., Zhang Y., Yan H., Sohail A., Zhao W., Wang D., Ma C., Cui L. (2025). Rapid and Preparative Separation of Polyphenols from Pods of *Paeonia suffruticosa* Andr. By Elution-Extrusion Counter-Current Chromatography Coupled with Inner-Recycling Mode. J. Sep. Sci..

[30] Lu Q., Gao Y., Xiang F., Zu Y., Zhang Y. (2015). Study on Extraction Process of Polysaccharides from *P. suffruticosa* Follicles. Bull. Bot. Res..

[31] Tan X., Zhou X., Chen H. (2017). Structure-activity relationship of plant polysaccharides. Zhongguo Zhong Yao Za Zhi = Zhongguo Zhongyao Zazhi = China J. Chin. Mater. Medica.

[32] Zou L., Wang C., Kuang X., Li Y., Sun C. (2016). Advance in flavonoids biosynthetic pathway and synthetic biology. China J. Chin. Mater. Medica.

[33] Zhong J., Li B., Jia Q., Li Y., Zhu W., Chen K. (2011). Advances in the structure-activity relationship study of natural flavonoids and its derivatives. Yao Xue Xue Bao = Acta Pharm. Sin..

[34] Xue-Xue W., Ai-Wu Y., Zhu-Ping T., Ying L., Can-Wei L., Meng-Ran F., Wei-Hong L., Peng-Fei G. (2020). Research progress on anti-alcoholic gastric injury active components and mechanisms of Chinese herbal medicine. Zhongguo Zhong Yao Za Zhi = Zhongguo Zhongyao Zazhi = China J. Chin. Mater. Medica.

[35] Yuan X., Chen J., Dai M. (2016). Paeonol promotes microRNA-126 expression to inhibit monocyte adhesion to ox-LDL-injured vascular endothelial cells and block the activation of the PI3K/Akt/NF-κB pathway. Int. J. Mol. Med..

[36] Min C., Liu H., Zhan F., Qiu W. (2009). Effect of paeonol on protecting endothelial cells of diabetic rats. Zhong Yao Cai = Zhongyaocai = J. Chin. Med. Mater..

[37] Hong X.F., Li L.I., Yang Z.X., Yan J. (2022). Paeoniflorin improves myocardial injury via inhibition of Src/VE-cadherin pathway in septic rats. Zhonghua Nei Ke Za Zhi.

[38] Wei Z., Tong D., Yang J., Zhao K., Meng X., Zhang Y. (2017). Action mechanism of total flavonoids of *Hippophae rhamnoides* in treatment of myocardial ischemia based on network pharmacology. Zhongguo Zhong Yao Za Zhi = Zhongguo Zhongyao Zazhi = China J. Chin. Mater. Medica.

[39] Li Y., Bao J., Xu J., Murad F., Bian K. (2010). Vascular dilation by paeonol—A mechanism study. Vasc. Pharmacol..

[40] Liu Y., Shao Q., Zhang H., Jia Y., Dai M. (2020). Inhibitory effect of paeonol on aortic endothelial inflammation in atherosclerotic rats by up-regulation of caveolin-1 expression and suppression of NF-κB pathway. Zhongguo Zhong Yao Za Zhi = Zhongguo Zhongyao Zazhi = China J. Chin. Mater. Medica.

[41] Tianjin Municipal Administration for Market Regulation and Quality Supervision (2018). Tianjin Municipal Standards for Processing of Chinese Materia Medica Decoction Pieces.

[42] Shanghai Municipal Medical Products Administration (2018). Shanghai Municipal Standards for Processing of Chinese Materia Medica Decoction Pieces.

[43] Jiangxi Provincial Food and Drug Administration (2008). Jiangxi Provincial Standards for Processing of Chinese Materia Medica Decoction Pieces.

[44] Henan Provincial Food and Drug Administration (2005). Henan Provincial Standards for Processing of Chinese Materia Medica Decoction Pieces.

[45] Sichuan Provincial Food and Drug Administration (2015). Sichuan Provincial Standards for Processing of Chinese Materia Medica Decoction Pieces.

[46] Chongqing Municipal Food and Drug Administration (2006). Chongqing Municipal Standards and Criteria for Processing of Chinese Materia Medica Decoction Pieces.

[47] Guizhou Provincial Medical Products Administration (2005). Guizhou Provincial Standards for Processing of Chinese Materia Medica Decoction Pieces.

[48] Shaanxi Provincial Medical Products Administration (2008). Shaanxi Provincial Standards for Chinese Materia Medica Decoction Pieces.

[49] Jiangsu Provincial Medical Products Administration (2002). Jiangsu Provincial Standards for Processing of Chinese Materia Medica Decoction Pieces.

[50] Zhejiang Provincial Food and Drug Administration (2015). Zhejiang Provincial Standards for Processing of Chinese Materia Medica.

[51] Anhui Provincial Medical Products Administration (2019). Anhui Provincial Standards for Processing of Chinese Materia Medica Decoction Pieces.

[52] Guangxi Zhuang Autonomous Region Food and Drug Administration (2007). Guangxi Zhuang Autonomous Region Standards for Processing of Chinese Materia Medica Decoction Pieces.

[53] Beijing Municipal Food and Drug Administration (2008). Beijing Municipal Standards for Processing of Chinese Materia Medica Decoction Pieces.

[54] Hunan Provincial Food and Drug Administration (2010). Hunan Provincial Standards for Processing of Chinese Materia Medica Decoction Pieces.

[55] Shandong Provincial Food and Drug Administration (2012). Shandong Provincial Standards for Processing of Chinese Materia Medica Decoction Pieces.

[56] Ningxia Hui Autonomous Region Medical Products Administration (2017). Ningxia Standards for Processing of Chinese Materia Medica Decoction Pieces.

[57] Hubei Provincial Medical Products Administration (2018). Hubei Provincial Standards for Processing of Chinese Materia Medica Decoction Pieces.

[58] Zhan L., Huang X., Ma J., Fang L., Ding Y., Luo Y. (2024). Study on Quality Standard of Carbonized Moutan Cortex. Chin. J. Ethnomedicine Ethnopharmacy.

[59] Xiao H., Zhao L., Yang X., Zhang Y., Lin F., Li J., Wang S., Gao J. (2022). HPLC-Based Fingerprint Analysis of *P. suffruticosa* Leaves and Establishment of Quantitative Method for 7 Components. J. Shanxi Med. Univ..

[60] Li Y., Li J., Xie Y., Luo H., Wu J., Lin F., Wang S. (2022). Determination of Cell Wall Breaking Rate and Analysis of Nutritional Components of Ultra-Fine Freeze-Dried Powder of *P. suffruticosa* Petals. J. Northwest Univ. (Nat. Sci. Ed.).

[61] Xiao H., Yang X., Luo H., Li Y., Li J., Lin F., Luo D., Wang S. (2021). Qualitative Identification and Content Determination of *P. suffruticosa* Petal Medicinal Materials. J. Shanxi Med. Univ..

[62] Wu J., Li J., Wang J., Li Y., Luo H., Lin F., Wang S. (2020). Determination of Wall Breaking Rate of *P. suffruticosa* Pollen and Content of Its 2 Monoterpene Glycosides. Northwest Pharm. J..

[63] Cao Y., Zhu Z., Guo Q., Liu L., Wang C. (2015). Study on Quality Grading Standard of Medicinal *P. suffruticosa* Seeds. China J. Chin. Mater. Medica.

[64] Yuan Q. (2022). Interpretation of National Standard “*P. suffruticosa* Seed Oil”. China Grain Econ..

[65] Yuan B., Gao G., Zhang H., Wu S., Lu L., Huang Y., Pan L. (2019). Study on the Connotation of Specifications and Grades of Moutan Cortex from Different Producing Areas. J. Anhui Agric. Sci..

[66] Liu J., Lu C., Feng R., Yang F., Yang Y. (2023). Simultaneous Determination of 7 Compounds in Moutan Cortex by Quantitative Analysis of Multi-Compounds by Single Marker (QAMS) Method. J. Chin. Med. Mater..

[67] Liu J., Li X., Bai H., Yang X., Mu J., Yan R., Wang S. (2023). Traditional uses, phytochemistry, pharmacology, and pharmacokinetics of the root bark of *Paeonia* × *suffruticosa* andrews: A comprehensive review. J. Ethnopharmacol..

[68] Jia S., Ji M., Di S.E., Huang S., Zeng R., Wang X. (2024). Content Determination of *P. suffruticosa* Leaf Extracts and Study on Their Antibacterial Activity Synergistic with Carboxymethyl Chitosan. World Notes Antibiot..

[69] Ning E., Chen L., Wang X., Zhang L., Wang F., Li X. (2023). Determination of Total Flavonoids, Total Phenols and Paeoniflorin in 20 Kinds of *P. suffruticosa* Leaves and Study on Their Antioxidant Activity. Feed Res..

[70] Niu X., Gao Y., Huai B., Yin E., Ma T., Li K., Huang L., Wang P., Hou M., Jiang W. (2021). Determination of Total Polysaccharide Content in Six Kinds of *P. suffruticosa* Leaves by Sulfuric Acid-Phenol Method. J. Shandong Agric. Eng. Univ..

[71] Dong L., Wang Y., Ning J., Zhang X., Ding W. (2020). Analysis and Evaluation of Nutritional Components of *P. suffruticosa* Leaves from Different Producing Areas in Heze. Sci. Technol. Food Ind..

[72] Wu X. (2017). Analysis of Nutritional Components in P. suffruticosa Leaves and Petals.

[73] Yan H., Wang Z., Chen Y., Jiang J., Cui L., Geng Y., Wang X. (2017). Study on Chemical Constituents of *Paeonia ostii* Petals. Shandong Sci..

[74] Su B., Sun Z., Zhang Y., Xin Z., Li H. (2017). Analysis of Polyphenol Compounds and Antioxidant Capacity of Petals from 11 P. suffruticosa Cultivars.

[75] Yu X., Zhang H., Wang J., Wang J., Wang Z., Li J. (2022). Phytochemical compositions and antioxidant activities of essential oils extracted from the flowers of *Paeonia delavayi* using supercritical carbon dioxide fluid. Molecules.

[76] Zhang X.X., Sun J.Y., Niu L.X., Zhang Y.L. (2017). Chemical compositions and antioxidant activities of essential oils extracted from the petals of three wild tree peony species and eleven cultivars. Chem. Biodivers..

[77] Sun S., Wang Y., Wang S., Wei L., Hu Y., Wang L. (2023). Analysis and Evaluation of Nutritional Components in Pollen and Anther Wall of *Paeonia ostii*. J. Nucl. Agric. Sci..

[78] Wang X., Shi X., Liu D., Xu X., Ma X., Shen W., Ma Q., Fan B. (2023). Chemical Constituents of Paeonia rockii Pollen and Its In Vitro Antioxidant Activity. Chin. Tradit. Pat. Med..

[79] Wang H., Yang L., Zu Y., Zhao X. (2014). Microwave-assisted simultaneous extraction of luteolin and apigenin from tree peony pod and evaluation of its antioxidant activity. Sci. World J..

[80] Zhang X., Ban Q., Wang X., Wang Z. (2018). Green and Efficient PEG-Based Ultrasonic-Assisted Extraction of Polysaccharides from Tree Peony Pods and the Evaluation of Their Antioxidant Activity In Vitro. BioMed Res. Int..

[81] Zhang L., Gong Q., Peng Y., Yue T. (2022). Optimization of Isolation Process and Characterization of Structural Properties of Cellulose from *P. suffruticosa* Follicles. J. Chongqing Norm. Univ. (Nat. Sci. Ed.).

[82] Lanzer P., Topol E.J. (2013). Pan Vascular Medicine: Integrated Clinical Management.

[83] Ge J. (2024). Chinese expert consensus on anti-thrombotic therapy for panvascular diseases (2023 edition). Cardiol. Plus.

[84] Hu Y., Zhao Y., Li P., Lu H., Li H., Ge J. (2023). Hypoxia and panvascular diseases: Exploring the role of hypoxia-inducible factors in vascular smooth muscle cells under panvascular pathologies. Sci. Bull..

[85] Xu R., Wang Z., Dong J., Yu M., Zhou Y. (2025). Lipoprotein (a) and panvascular disease. Lipids Health Dis..

[86] Yu Y., Cai Y., Yang F., Bai R., Shi D. (2025). Preliminary Exploration of TCM Pathogenesis of panvascular Diseases Based on the “Stasis-Toxin” Theory. China J. Chin. Mater. Medica.

[87] Dong S., Zhang F., Cheng S. (2025). Discussion on Prevention and Treatment of panvascular Diseases Based on the “Stasis-Turbidity Damaging Vessels” Theory. Jiangxi J. Tradit. Chin. Med..

[88] Wang J., Xia T. (2025). Accountability in AI medicine: A critical appraisal of ChatGPT in patient self-management and screening. Clin. Mol. Hepatol..

[89] Guo W., Yi X. (2025). Advancements and future prospects in the study of panvascular disease. Clin. Hemorheol. Microcirc..

[90] Wang J., Li J., Dong Y., Chen C., Liu Y., Liu C., Liu L., Sun X. (2025). TCM Connotation and Prevention Strategies of panvascular Diseases. Chin. J. Exp. Tradit. Med. Formulae.

[91] Xie J., Cui Y., Geng B., Tang C., Zeng Q. (2014). The antihypertensive effect of adrenomedullin 2 and related mechanism. Zhongguo Ying Yong Sheng Li Xue Za Zhi = Zhongguo Yingyong Shenglixue Zazhi = Chin. J. Appl. Physiol..

[92] Xu S., Ilyas I., Little P.J., Li H., Kamato D., Zheng X., Luo S., Li Z., Liu P., Han J. (2021). Endothelial dysfunction in atherosclerotic cardiovascular diseases and beyond: From mechanism to pharmacotherapies. Pharmacol. Rev..

[93] Wei S., Evans P.C., Strijdom H., Xu S. (2025). HIV Infection, Antiretroviral Therapy and Vascular Dysfunction: Effects and Mechanisms. Pharmacol. Res..

[94] Sun Y., He W., Huang Y., Song X., Li S. (2025). Research Progress on Vascular Injury-Related Markers. Chin. J. Mol. Cardiol..

[95] Li Y., Liu J., Dang A. (2023). Research Progress on Vascular Endothelial Dysfunction and Atherosclerosis. Chin. J. Hypertens..

[96] Feng X.H., Fan T.F., Hou Y.F., Guo W.J., Gao R., Wang J. (2023). Research advances of inflammatory cells and aortic intrinsic cells in the pathogenesis of aortic dissection. Zhonghua Xin Xue Guan Bing Za Zhi.

[97] Badimon L., Vilahur G. (2014). Thrombosis formation on atherosclerotic lesions and plaque rupture. J. Intern. Med..

[98] Skrzypczak-Wiercioch A., Sałat K. (2022). Lipopolysaccharide-induced model of neuroinflammation: Mechanisms of action, research application and future directions for its use. Molecules.

[99] Guo W., Yan S., Zhao G. (2024). Upregulated ATF1 promotes Lipopolysaccharide Induced Inflammatory Response and inhibits osteogenic differentiation of Human Periodontal Ligament cells by regulating NF-kappaB pathway. Discov. Med..

[100] Li M., Sun L., Niu X., Chen X., Tian J., Kong Y., Wang G. (2019). Astaxanthin protects lipopolysaccharide-induced inflammatory response in Channa argus through inhibiting NF-κB and MAPKs signaling pathways. Fish Shellfish Immunol..

[101] Blokhina O., Virolainen E., Fagerstedt K.V. (2003). Antioxidants, oxidative damage and oxygen deprivation stress: A review. Ann. Bot..

[102] Jomova K., Raptova R., Alomar S., Alwasel S., Nepovimova E., Kuca K., Valko M. (2023). Reactive oxygen species, toxicity, oxidative stress, and antioxidants: Chronic diseases and aging. Arch. Toxicol..

[103] Afanas Ev I. (2011). ROS and RNS signaling in heart disorders: Could antioxidant treatment be successful?. Oxidative Med. Cell. Longev..

[104] Kattoor A.J., Pothineni N.V.K., Palagiri D., Mehta J.L. (2017). Oxidative stress in atherosclerosis. Curr. Atheroscler. Rep..

[105] Urso C., Caimi G. (2011). Oxidative stress and endothelial dysfunction. Minerva Medica.

[106] Liu X., Chen K., Wang Y., Wang J., Wang C. (2025). Association between remnant cholesterol and atherosclerosis plaques in single and multiple vascular territories. Zhong Nan Da Xue Xue bao. Yi Xue Ban = J. Cent. South Univ. Med. Sci..

[107] Hartley A., Haskard D., Khamis R. (2019). Oxidized LDL and anti-oxidized LDL antibodies in atherosclerosis–novel insights and future directions in diagnosis and therapy. Trends Cardiovasc. Med..

[108] Mao Q., Xiang C., Tong W., Zhao X. (2020). Correlation between Serum Klotho and Glucose-Lipid Metabolism Disorder in Patients with Coronary Atherosclerosis. J. Clin. Cardiol..

[109] Piao F., Zhou J. (2025). Effect of circTLK1 Targeting MicroRNA-424-5p on Oxidized Low-Density Lipoprotein-Induced Injury of Human Coronary Artery Endothelial Cells. Prev. Treat. Cardio-Cereb. Vasc. Dis..

[110] Yin C. (2021). Study on the Effect and Mechanism of Exercise-Based Cardiac Rehabilitation on Platelet Function in Patients with Coronary Heart Disease.

[111] Zhou X.H., Cheng Z.P., Hu Y. (2019). Platelet GP I b- IX—V receptor-mediated mechanism and its application in thrombotic diseases. Zhonghua Xue Ye Xue Za Zhi = Zhonghua Xueyexue Zazhi.

[112] Li J., Liang G., Wang S., Li S., Ke H. (2022). Correlation between Changes of Serum Inflammatory Factors and Vascular Endothelial Injury Markers in Left Atrium and Thrombosis in Patients with Atrial Fibrillation. Chin. Gen. Pract..

[113] Duan Y., Yh D.U., Liu H. (2021). Research advances of occludin in vascular endothelial injury. Sheng Li Xue Bao [Acta Physiol. Sin.].

[114] Zhou M., Yu Y., Zhao Y., Luo X., Zhu J., Hu Y., Jian W. (2023). Research progress in targeting autophagy of traditional Chinese medicine and natural compounds to regulate atherosclerosis. Zhongguo Zhong Yao Za Zhi = Zhongguo Zhongyao Zazhi = China J. Chin. Mater. Medica.

[115] Xi Y., Huang Y., Du R., Wang Y., Wang G., Yin T. (2018). Endothelial injury and its repair strategies after intravascular stents implantation. Sheng Wu Yi Xue Gong Cheng Xue Za Zhi = J. Biomed. Eng. = Shengwu Yixue Gongchengxue Zazhi.

[116] Yoo M.Y., Lee B.H., Choi Y.H., Lee J.W., Seo J.H., Oh K.S., Koo H.N., Seo H.W., Yon G.H., Kwon D.Y. (2006). Vasorelaxant effect of the rootbark extract of *Paeonia moutan* on isolated rat thoracic aorta. Planta Medica.

[117] Ha D.T., Ngoc T.M., Lee I., Lee Y.M., Kim J.S., Jung H., Lee S., Na M., Bae K. (2009). Inhibitors of aldose reductase and formation of advanced glycation end-products in Moutan Cortex (*Paeonia suffruticosa*). J. Nat. Prod..

[118] Furuya R., Hu H., Zhang Z., Shigemori H. (2012). Suffruyabiosides A and, B.; two new monoterpene diglycosides from moutan cortex. Molecules.

[119] Ha D.T., Trung T.N., Hien T.T., Dao T.T., Yim N., Ngoc T.M., Oh W.K., Bae K. (2010). Selected compounds derived from Moutan Cortex stimulated glucose uptake and glycogen synthesis via AMPK activation in human HepG2 cells. J. Ethnopharmacol..

[120] Ha D.T., Tuan D.T., Thu N.B., Nhiem N.X., Ngoc T.M., Yim N., Bae K. (2009). Palbinone and triterpenes from Moutan Cortex (*Paeonia suffruticosa*, Paeoniaceae) stimulate glucose uptake and glycogen synthesis via activation of AMPK in insulin-resistant human HepG2 Cells. Cheminform.

[121] Xiao H., Yao R., Liu B., Duan L., Liu J., Lin F., Wang S., Gao J. (2025). Comparative evaluation of moutan pods and moutan barks by HPLC-DAD-ESI-MS/MS technique. Sci. Rep..

[122] Sajadimajd S., Deravi N., Forouhar K., Rahimi R., Kheirandish A., Bahramsoltani R. (2023). Endoplasmic reticulum as a therapeutic target in type 2 diabetes: Role of phytochemicals. Int. Immunopharmacol..

[123] Yang X., Xue X., He Y., Song P., Guo L., Hou X. (2024). Exploring the Effect of Active Components in Oil Tree Peony Seed Meal on Swine Disease Resistance and its Potential Mechanisms Based on Network Pharmacology and Molecular Docking. Chem. Biodivers..

[124] Zhu X., Fang Z. (2014). New monoterpene glycosides from the root cortex of *Paeonia suffruticosa* and their potential anti-inflammatory activity. Nat. Prod. Res..

[125] Ding L., Zhao F., Chen L., Jiang Z., Liu Y., Li Z., Qiu F., Yao X. (2012). New monoterpene glycosides from *Paeonia suffruticosa* Andrews and their inhibition on NO production in LPS-induced RAW 264.7 cells. Bioorg. Med. Chem. Lett..

[126] Lee S., Lee I., Mar W. (2003). Inhibition of inducible nitric oxide synthase and cyclooxygenase-2 activity by 1,2,3,4,6-penta-O-galloyl-β-D-glucose in murine macrophage cells. Arch. Pharmacal Res..

[127] Choi Y.H., Yoo H.J., Noh I.C., Lee J.M., Park J.W., Choi W.S., Choi J.H. (2012). Bioassay-guided isolation of novel compound from *Paeonia suffruticosa* Andrews roots as an IL-1β inhibitor. Arch. Pharmacal Res..

[128] Gao J., Wang L., Zhao C., Wu Y., Lu Z., Gu Y., Ba Z., Wang X., Wang J., Xu Y. (2021). Peony seed oil ameliorates neuroinflammation-mediated cognitive deficits by suppressing microglial activation through inhibition of NF-κB pathway in presenilin 1/2 conditional double knockout mice. J. Leukoc. Biol..

[129] Deng R., Gao J., Yi J., Liu P. (2022). Could peony seeds oil become a high-quality edible vegetable oil? The nutritional and phytochemistry profiles, extraction, health benefits, safety and value-added-products. Food Res. Int..

[130] Lv M., Yang Y., Choisy P., Xu T., Pays K., Zhang L., Zhu J., Wang Q., Li S., Wang L. (2023). Flavonoid components and anti-photoaging activity of flower extracts from six Paeonia cultivars. Ind. Crop. Prod..

[131] Lu Y., Liu W., Zhang M., Deng Y., Jiang M., Bai G. (2019). The screening research of NF-κB inhibitors from *Moutan cortex* based on bioactivity-integrated UPLC-Q/TOF-MS. Evid. Based Complement. Altern. Med..

[132] Luo L., Wu S., Chen R., Rao H., Peng W., Su W. (2020). The study of neuroprotective effects and underlying mechanism of *Naoshuantong capsule* on ischemia stroke mice. Chin. Med..

[133] Matsuda H., Ohta T., Kawaguchi A., Yoshikawa M. (2001). Bioactive Constituents of Chinese Natural Medicines. VI. Moutan Cortex. (2): Structrues and Radical Scavenging Effects of Suffruticosides A, B, C, D, and E, and E and Galloyl-oxypaeoniflorin. Chem. Pharm. Bull..

[134] An R., Kim H., Lee S., Jeong G., Sohn D., Park H., Kwon D., Lee J., Kim Y. (2006). A new monoterpene glycoside and antibacterial monoterpene glycosides from *Paeonia suffruticosa*. Arch. Pharmacal Res..

[135] Ding L., Jiang Z., Liu Y., Chen L., Zhao Q., Yao X., Zhao F., Qiu F. (2012). Monoterpenoid inhibitors of NO production from *Paeonia suffruticosa*. Fitoterapia.

[136] Ryu G., Park E.K., Joo J.H., Lee B.H., Choi B.W., Jung D.S., Lee N.H. (2001). A new antioxidant monoterpene glycoside, α-benzoyloxypaeoniflorin from *Paeonia suffruticosa*. Arch. Pharmacal Res..

[137] Xiao C., Wu M., Chen Y., Jia P., Jia R., Zheng X. (2014). Metabolomic analysis provides novel chemotaxonomic characteristics for phenotypic cultivars of tree peony. Anal. Methods.

[138] Ding L., Qiu T., Liu Z., Chen L., Oppong M., Zhang D., Zhang B., Bai G., Qiu F. (2017). Systematic characterization of the metabolites of paeonol in rats using ultra performance liquid chromatography coupled with electrospray ionization quadrupole time-of-flight tandem mass spectrometry with an integrative strategy. J. Chromatogr. B.

[139] Jang M.H., Kim K.Y., Song P.H., Baek S.Y., Seo H.L., Lee E.H., Lee S.G., Park K.I., Ahn S.C., Kim S.C. (2017). Moutan Cortex Protects Hepatocytes against Oxidative Injury through AMP-Activated Protein Kinase Pathway. Biol. Pharm. Bull..

[140] Wu G., Shen Y., Nie R., Li P., Jin Q., Zhang H., Wang X. (2020). The bioactive compounds and cellular antioxidant activity of Herbaceous peony (*Paeonia lactiflora* Pall) seed oil from China. J. Food Sci..

[141] Pagliarulo C., Sansone F., Moccia S., Russo G.L., Aquino R.P., Salvatore P., Stasio M.D., Volpe M.G. (2016). Preservation of strawberries with an antifungal edible coating using peony extracts in chitosan. Food Bioprocess Technol..

[142] Zhang X., Shi Q., Ji D., Niu L., Zhang Y. (2017). Determination of the phenolic content, profile, and antioxidant activity of seeds from nine tree peony (*Paeonia section* Moutan DC.) species native to China. Food Res. Int..

[143] Wu Y.Q., Wei M.R., Zhao D.Q., Tao J. (2016). Flavonoid content and expression analysis of flavonoid biosynthetic genes in herbaceous peony (*Paeonia lactiflora* Pall.) with double colors. J. Integr. Agric..

[144] Tang Y., Huang M., Zhang Y. (2012). Comparison of in vitro anti-oxidative activities among Siwu Decoction Serial Recipes, their composed crude herbs, and main aromatic acids, as well as their dose-effect correlation. Zhongguo Zhong Xi Yi Jie He Za Zhi Zhongguo Zhongxiyi Jiehe Zazhi = Chin. J. Integr. Tradit. West. Med..

[145] Yin D., Li S., Shu Q., Gu Z., Wu Q., Feng C., Xu W., Wang L. (2018). Identification of microRNAs and long non-coding RNAs involved in fatty acid biosynthesis in tree peony seeds. Gene.

[146] Lin H.C., Ding H.Y., Ko F.N., Teng C.M., Wu Y.C. (1999). Aggregation inhibitory activity of minor acetophenones from Paeonia species. Planta Medica.

[147] Li G., Seo C.S., Lee K.S., Kim H.J., Chang H.W., Jung J.S., Song D.K., Son J.K. (2004). Protective constituents against sepsis in mice from the root cortex of *Paeonia suffruticosa*. Arch. Pharmacal Res..

[148] Ding H., Wu Y., Lin H., Chan Y., Wu P., Wu T. (1999). Glycosides from *Paeonia suffruticosa*. Chem. Pharm. Bull..

[149] Ding H.Y., Lin H.C., Teng C.M., Wu Y.C. (2000). Phytochemical and pharmacological studies on Chinese Paeonia species. J. Chin. Chem. Soc..

